# *Bacillus thuringiensis* pathogenicity islands encode regulatory circuits controlling insecticidal Cry toxin expression during vegetative growth

**DOI:** 10.1371/journal.ppat.1014476

**Published:** 2026-07-29

**Authors:** Bowen Zhang, Zhiyu Liu, Hang Zhang, Alejandra Bravo, Mario Soberón, Jinshui Zheng, Ming Sun, Kai Wang, Fuping Song, Donghai Peng

**Affiliations:** 1 National Key Laboratory of Agricultural Microbiology, Huazhong Agricultural University, Wuhan, Hubei, China; 2 College of Life Science and Technology, Huazhong Agricultural University, Wuhan, China; 3 Instituto de Biotecnología, Universidad Nacional Autónoma de México, Cuernavaca, Morelos, Mexico; 4 College of Informatics, Huazhong Agricultural University, Wuhan, China; 5 State Key Laboratory for Biology of Plant Diseases and Insect Pests, Institute of Plant Protection, Chinese Academy of Agricultural Sciences, Beijing, China; University of Massachusetts Chan Medical School, UNITED STATES OF AMERICA

## Abstract

*Bacillus thuringiensis* (Bt) produces insecticidal toxins, including Cry and Vip3 proteins, that are widely used for biological pest control. Cry proteins are classically expressed during sporulation under the control of sporulation-specific σ factors, whereas Vip3 is produced during vegetative growth, suggesting distinct regulatory pathways. Notably, many *cry* and *vip3A* genes are clustered within pathogenicity islands (PAIs), such as BtPAI-1. However, whether these PAIs also encode regulatory mechanisms coordinating toxin expression remains unclear. Here, we identify VipR, a BtPAI-1-encoded transcriptional regulator, as an activator of insecticidal gene expression during the vegetative phase in Bt strains HD-1 and CT-43. In these strains, VipR promotes the transcription of BtPAI-1 associated insecticidal genes, including *vip3A* and selected *cry* genes*,* resulting in premature Cry protein accumulation and increased insecticidal activity. In addition, VipR contributes to the vegetative-phase expression of the non-BtPAI-1 *cry9Aa* genes in strain BGSC 4AE1. Phylogenetic analysis revealed that *vipR* is widely distributed in one-third of Bt strains, and is strongly associated with PAIs. Futhermore, heterologous expression of *vipR* in BGSC 4J5 and HD-73 was sufficient to activate vegetative-phase transcription of some *cry* independently of sporulation-specific σ factor cascade. These results support a role for VipR in coordinating vegetative-phase expression of insecticidal genes in the Bt strains examined and suggest that BtPAI-1 can encode both insecticidal determinants and regulatory functions that influence their expression. These findings provide new insights into the regulatory architecture of Bt pathogenicity islands and may facilitate the engineering of strains with enhanced insecticidal activity.

## Author summary

*Bacillus thuringiensis* (Bt) is widely used as a biological pesticide because it produces insecticidal proteins that kill insect larvae. These proteins are generally produced at different stages of bacterial growth: Cry proteins during sporulation and Vip3 proteins during vegetative growth. Many of these genes encoding these toxins are located within mobile DNA regions called pathogenicity islands, such as BtPAI-1, but it has been unclear whether these regions also contain regulators that control their expression.

Here, we investigated the function of VipR, a transcriptional regulator encoded within BtPAI-1. Using the Bt strains HD-1 and CT-43, we found that VipR activates vegetative-phase expression of *vip3A* and selected *cry* genes located in the same BtPAI-1 island, resulting in earlier accumulation of Cry proteins and increased insecticidal activity. We also show that expression of VipR in strain BGSC 4J5, HD-73 and BGSC 4AE1 promotes vegetative-phase transcription of *cry* genes independently of the sporulation regulatory cascade. These findings uncover a previously unrecognized regulatory mechanism controlling the timing of toxin production and show that BtPAIs can encode both toxin genes and regulatory functions that coordinate their expression.

## Introduction

The evolution of bacterial virulence is shaped by long-term interactions with hosts and environmental pressures, during which virulence phenotypes are continuously optimized through genetic variation and natural selection [1–5]. Throughout this process, functionally related virulence genes are frequently clustered within specific genomic regions by horizontal gene transfer (HGT) of large mobile genetic elements, known as pathogenicity islands (PAIs) [6,7]. Gene expression within PAIs is typically regulated by both host-encoded regulatory networks and PAI-encoded regulatory factors, enabling precise temporal and spatial control of virulence factor production during infection [8–11]. For example, in *Staphylococcus aureus*, the *enterotoxin* genes located in the core pathogenicity island SaPI are coordinately regulated by the host-encoded quorum-sensing transcription factor Agr and the SaPI-encoded repressor Stl [12–15]. Such dual-layer regulatory architecture ensures both accurate and coordinated expression of virulence determinants while integrating horizontally acquired genes into host regulatory networks [8].

*Bacillus thuringiensis* (Bt) is a globally important biocontrol bacterium widely used to manage agricultural and forestry pests, including insects from the orders Lepidoptera, Coleoptera and nematodes [16,17]. Its insecticidal activity is primarily mediated by plasmid-encoded toxins, most notably three-domain crystal proteins (Cry) and vegetative insecticidal proteins (Vip3) [16–18]. These insecticidal protein genes are clustered within plasmid-borne pathogenicity islands (PAIs) [19]. Previous studies have shown that the expression of these insecticidal proteins is tightly regulated in a growth phase-dependent manner. The transcription of most core toxin genes, including members of the *cry1* and *cry4* families, is dependent on the sporulation-specific σ^E^/σ^K^ regulatory cascade [20–22] and additional regulatory factors such as *nprR*, resulting in substantial Cry protein accumulation primarily during the late growth or sporulation stages [23,24]. In contrast, a limited subset of insecticidal genes, such as certain members of the *vip3* [25] and *cry3* families [26], are expressed during the vegetative phase [27–29]. This temporal regulation restricts most Cry protein production to late growth phases, thereby limiting both the timing and extent of toxin accumulation. However, previous studies have demonstrated that replacing the native sporulation-dependent promoters of *cry* genes with vegetative-phase-active promoters drives the premature expression of Cry proteins and markedly enhances insecticidal activity [30,31]. In addition, the transcription factor CpcR in the Bt LM1212 strain specifically activates the vegetative-phase expression of sporulation-dependent *cry* genes (including *cry1A* and *cry35-like*) in a non-sporulating subpopulation during growth, whereas crystal proteins are not produced in the sporulating cells [32,33]. These findings suggest that endogenous regulatory pathways may enable earlier activation of Cry protein production.

Multiple insecticidal protein genes are encoded in BtPAIs, but it is not known if these genetic regions encode additional native regulatory elements for Cry proteins. Despite extensive characterization of sporulation-dependent regulation of insecticidal genes in Bt*,* it remains unclear whether Bt harbors endogenous regulatory mechanisms that enable toxin gene expression during the vegetative phase. In particular, the potential contribution of BtPAIs to the temporal control of insecticidal gene expression has not been systematically explored.

Previous studies identified that the transcriptional regulator *vipR* is located upstream of several insecticidal toxin genes, including *vip3A*, *cry2Ab* and *cry1Aa*, within the pathogenicity island 1 (BtPAI-1) on the large plasmid pBMB299 of the highly virulent Bt HD-1 strain [34]. VipR has been shown to positively regulate *vip3Aa* expression during vegetative growth [34]. Notably, bioinformatic analyses have identified potential VipR-binding motifs within the promoter regions of several *cry* genes also located in the same BtPAI-1 [34], suggesting that VipR may function as a broader regulator, coordinating the expression of multiple toxin genes within this PAI.

Here, we demonstrate that VipR can directly activate the transcription of different insecticidal genes including *cry1Aa*, *cry2Aa*, *cry2Ab* and *cry1Ia* within BtPAI-1 during vegetative growth. This VipR-dependent temporal transcriptional activation reprogramming results in premature Cry protein accumulation, enhancing the insecticidal activity. Phylogenetic analyses revealed that *vipR* is present in approximately 33% of Bt strains and is widely associated with pathogenicity islands. Moreover, heterologous expression of *vipR* significantly upregulates insecticidal protein genes containing the predicted VipR-binding motifs. These findings reveal that VipR functions as an endogenous transcriptional regulator embedded within BtPAI-1, which enables vegetative-phase activation of *cry1Aa*, *cry2Aa*, *cry2Ab* and *cry1Ia* genes present in this PAI, thereby challenging the classical view that their expression is strictly dependent on sporulation-specific regulatory signals. More broadly, our results suggest that Bt pathogenicity islands, such as BtPAI-1, may disseminate not only toxin genes but also regulatory circuits that contribute to coordinating their expression.

## Results

### VipR positively regulates *cry* genes expression during the vegetative phase

Previous studies have reported that VipR positively regulates expression of the *vip3Aa* located in BtPAI-1 during the vegetative phase [34]. Further bioinformatic analysis also identified putative VipR-binding motifs in the promoter regions of several *cry* genes located within the same pathogenicity island [34], suggesting that VipR may be involved in regulating their expression. Based on this, we hypothesized that VipR may regulate the expression of *cry* genes on BtPAI-1. Therefore, we constructed a *vipR* knockout mutant (HD-1Δ*vipR*) in Bt subsp. *kurstaki* strain HD-1 (S1A and S1B Fig). Growth curve analyses showed no significant differences in growth dynamics between HD-1 and HD-1Δ*vipR* (S1C Fig), indicating that deletion of *vipR* does not affect bacterial growth.

**Fig 1:**
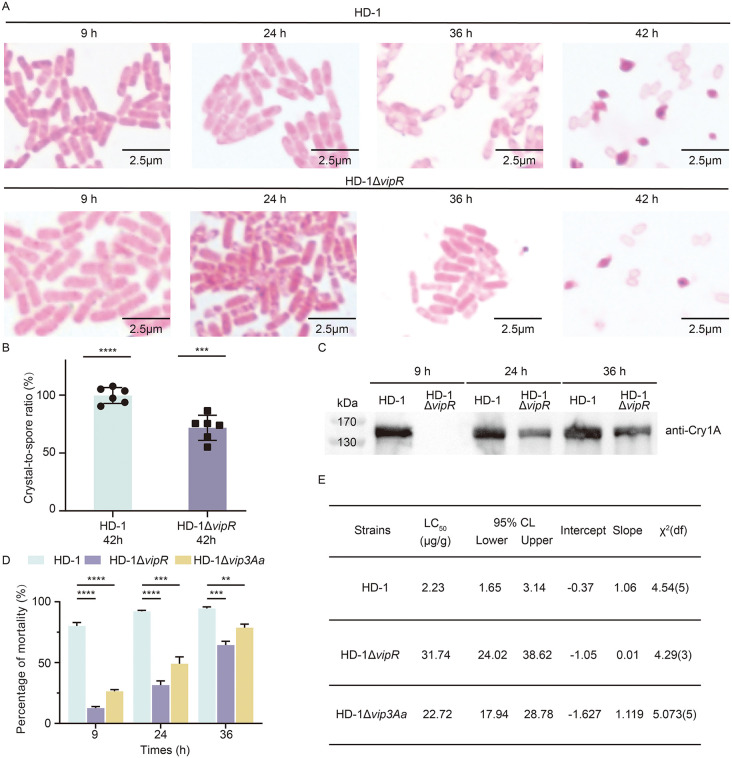
VipR promotes early Cry proteins accumulation during vegetative growth and enhances insecticidal activity of Bt. **(A)** Representative microscopic images of HD-1 and HD-1Δ*vipR* cells at 9 h, 24 h, 36 h and 42 h of growth. The images shown are 4 × enlarged views of the original micrographs, and the corresponding original images are provided in S2 Fig. Scale bar = 2.5 μm. **(B)** Crystal-to-spore ratio in HD-1 and HD-1Δ*vipR* at 42 h. The ratios were determined by counting crystals and spores in six randomly selected microscopic fields. Data represent the means ± SD (n = 6 biological replicates). Statistical procedures are specified in Materials and methods. ***P < 0.001, ****P < 0.0001. **(C)** Western blot detection of Cry1A protein in HD-1 and HD-1Δ*vipR* strains at 9 h, 24 h and 36 h. Cry1A was detected using an anti-Cry1A primary antibody and IRDye 680-conjugated goat anti-rabbit secondary antibody. All protein samples were extracted from an identical number of cells (1 × 10^7^ colony-forming units (CFU)/mL), and 20 µL of each sample (containing approximately 2 × 10^5^ CFU equivalents) was loaded per lane. **(D)** Larval mortality of *Spodoptera frugiperda* larvae after exposure to fermentation broth samples collected from HD-1, HD-1Δ*vip3Aa* and HD-1Δ*vipR* cultures at different growth phases. The fermentation broth samples were not lyophilized; fresh cultures were used directly. Prior to the bioassay, the cell concentration was determined using the dilution plate technique, and all samples were adjusted to the same cell density (1 × 10^7^ CFU/mL) with sterile ddH_2_O and then further diluted 4-fold (2.5 × 10^6^ CFU/mL). Subsequently, 3 mL of each diluted sample was mixed with 15 g of artificial diet, resulting in a final concentration of 5 × 10^5^ CFU per gram of diet, and the mixture was aliquoted into 24-well plates (one larva per well). Data represent the means ± SD (n = 3 biological replicates). **P < 0.01, ***P < 0.001, ****P < 0.0001. **(E)** Comparison of LC_50_ values of spore-crystal mixtures from HD-1, HD-1Δ*vip3Aa* and HD-1Δ*vipR* strains against *S. frugiperda*. Prior to LC_50_ determination, the cell concentration in each fermentation broth sample was determined using the dilution plate technique, and all samples were adjusted to the same cell density (1 × 10^7^ CFU/mL) with sterile ddH_2_O. The normalized samples were then lyophilized. The lyophilized spore-crystal powders were resuspended in ddH_2_O and then serially diluted from 1000 μg/mL to 100, 10, 5, 2.5, 1.25, and 0.625 μg/mL. 3 mL of each dilution was mixed with 15 g of artificial diet, resulting in final concentrations of 200, 20, 2, 1, 0.5, 0.25, and 0.125 μg per gram of diet. LC_50_ is the protein concentration causing 50% mortality of target insects, expressed as μg lyophilized spore-crystal mixture per gram of diet. The LC_50_ values include their corresponding 95% confidence limits (95% CL), intercept, slope, and Chi-square (χ^2^) statistics are shown.

Parasporal crystal formation was then examined at different growth stages by microscopy observations. At the vegetative phase (9 h), no obvious spores were observed in either strain. However, large fuchsin-stained inclusion bodies were readily detected in HD-1, whereas only small inclusion-like structures were observed in a subset of HD-1Δ*vipR* cells (Figs 1A and S2). At the early (24 h) and mid (36 h) sporulation phases, both HD-1 and HD-1Δ*vipR* began to form spores and visible parasporal crystal inclusions. However, spores and crystals remained closely associated and could not be reliably distinguished for quantitative analysis at these stages. At the late sporulation phase (42 h), spores were almost completely formed in both strains. However, the crystal-to-spore ratio remained significantly higher in HD-1 (99.6% ± 6.3%) than in HD-1Δ*vipR* (71.7% ± 8.0%) (Fig 1A and 1B).

To validate these phenotypic differences at the protein level, Cry1Aa production was analyzed by western blot. Cry1Aa was clearly detected in HD-1 during the vegetative phase (9 h) but was undetectable in HD-1Δ*vipR* (Fig 1C). Upon entry into the sporulation phase (24–36 h), Cry1Aa expression was detected in both strains after normalization to equivalent cell numbers. Analysis of these data showed higher protein levels of Cry1Aa in HD-1 than in HD-1Δ*vipR* (Fig 1C). To determine whether the reduced Cry accumulation in the *vipR* mutant affected insecticidal activity, bioassays against *Spodoptera frugiperda* larvae were performed. Because VipR positively regulates *vip3Aa* expression [34] and Vip3Aa represents a major vegetative-phase insecticidal protein, a *vip3Aa* deletion mutant (HD-1Δ*vip3Aa*) was constructed in the HD-1 background as a control strain to distinguish the respective contributions of Vip3Aa and Cry proteins in toxicity against *S. frugiperda* (S3 Fig). The insecticidal activities of HD-1, HD-1Δ*vipR* and HD-1Δ*vip3Aa* were evaluated at different growth phases. The results showed that during the vegetative phase (9 h), the fermentation broth of HD-1 caused 80.1% ± 2.9% larval mortality, whereas that of HD-1Δ*vipR* caused almost 6.4-fold lower values with 12.5% ± 1.4% mortality (Fig 1D). Notably, although the insecticidal activity of HD-1Δ*vip3Aa* at 9 h (26.4% ± 1.4%) remained significantly lower than that of wild-type HD-1, it was substantially higher than that of HD-1Δ*vipR*. These results indicate that the severe reduction in early insecticidal activity caused by *vipR* deletion cannot be explained solely by the loss of *vip3Aa*, but is also associated with the reduced accumulation of Cry proteins during the vegetative phase. During the sporulation phase (24 h and 36 h), HD-1 caused mortality rates of 92.1% ± 0.8% and 94.4% ± 1.4%, respectively, whereas HD-1Δ*vipR* exhibited substantially reduced mortalities of 31.5% ± 3.5% and 64.4% ± 3.2%, respectively (Fig 1D). However, the insecticidal activity of the HD-1Δ*vip3Aa* fermentation broth at the sporulation phases (24 h and 36 h) remained significantly lower than that of HD-1 but higher than that of HD-1Δ*vipR*, with mortality rates of 49.1% ± 5.6% and 78.7% ± 2.9%, respectively. These results suggest that the reduced insecticidal activity observed in HD-1Δ*vipR* during sporulation cannot be explained solely by the loss of Vip3Aa. Rather, it is also attributable to the reduced accumulation of Cry proteins caused by *vipR* deletion.

Consistent with these results, determination of the median lethal concentration (LC_50_) using spore-crystal mixtures showed that the LC_50_ value of HD-1 was 2.23 μg/g (with 95% confident values of 1.65-3.14 μg/g), whereas HD-1Δ*vipR* displayed a significantly higher LC_50_ value of 31.74 μg/g (with 95% confidence values of 24.02-38.62 μg/g). The absence of overlapping in the 95% confidence values confirmed a 15-fold reduction in toxicity of HD-1Δ*vipR* compared with HD-1 (Fig 1E). The HD-1Δ*vip3Aa* strain exhibited an intermediate LC_50_ value of 22.72 μg/g (with 95% confidence limits of 17.94-28.78 μg/g), representing an approximately 10-fold reduction in toxicity compared with HD-1, consistent with previous reports describing the contribution of Vip3Aa to insecticidal activity [35]. Although HD-1Δ*vip3Aa* exhibited higher toxicity than HD-1Δ*vipR*, the overlap between their 95% confidence intervals indicated that this difference was not statistically significant. This may be due to partial compensation by the substantial Cry protein production during the late sporulation phase.

Collectively, these results demonstrate that VipR not only regulates Vip3Aa expression but also promotes Cry protein production during vegetative growth. Also, the premature expression of Cry proteins significantly contributes to the insecticidal activity of the Bt HD-1 strain.

### VipR directly binds to the promoters of *cry* genes in BtPAI-1 and activates their expression during vegetative growth

**Fig 2:**
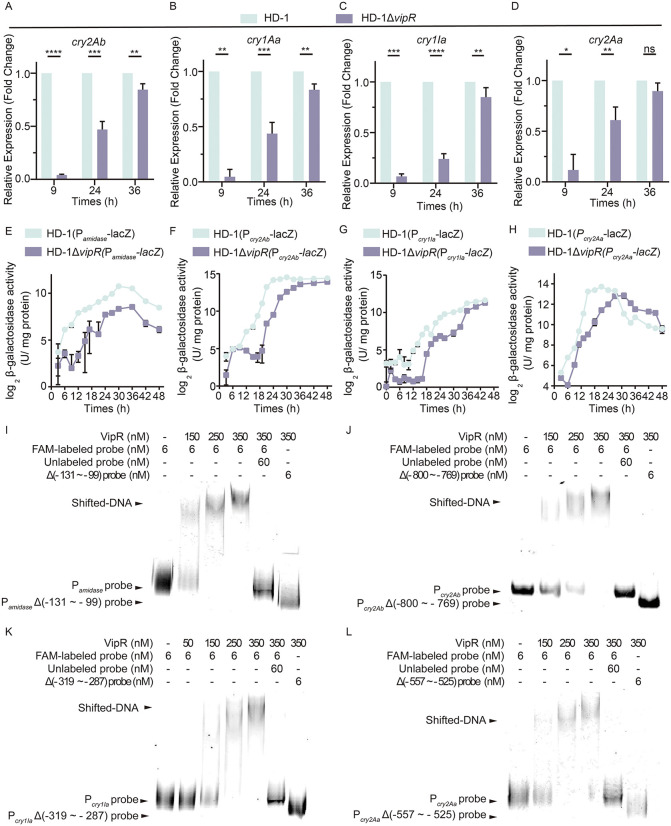
VipR directly activates transcription of multiple *cry* genes within BtPAI-1 by binding to their promoter regions. **(A–D)** Relative transcriptional levels of *cry2Ab* **(A)**, *cry1Aa* **(B)**, *cry1Ia* **(C)**, and *cry2Aa* **(D)** in HD-1 and HD-1Δ*vipR* at 9 h, 24 h, and 36 h, determined by quantitative reverse transcription PCR (qRT-PCR). The *gapdh* and *16S rRNA* were used as internal reference genes. Data represent the means ± SD (n = 3 biological replicates). Statistical significance was determined as described in Materials and methods. ns, *P* > 0.05; **P < 0.01; ***P < 0.001; ****P < 0.0001. **(E-H)** β-galactosidase reporter assays showing promoter activities of *amidase* **(E)**, *cry2Ab* **(F)**, *cry1Ia* **(G)**, and *cry2Aa* **(H)** in HD-1 and HD-1Δ*vipR*. Promoter regions were fused to a *lacZ* reporter gene, and β-galactosidase activity was measured at the indicated time points. Data represent the means ± SD from three independent experiments. Different letters indicate significant differences between groups (*P* < 0.05). **(I–L)** Electrophoretic mobility shift assays (EMSA) demonstrating direct binding of VipR to the promoter regions of *amidase* **(I)**, *cry2Ab* **(J)**, *cry1Ia* **(K)**, and *cry2Aa* **(L)**. FAM-labeled DNA promoter probes (P_*amidase*_-FAM, P_*cry2Ab*_-FAM, P_*cry1Ia*_-FAM and P_*cry2Aa*_-FAM), were incubated with increasing concentrations of purified recombinant VipR protein. To exclude non-specific binding, 100 ng of poly(dI-dC) was added to each reaction mixture. For competition assays, a 10-fold molar excess of unlabeled specific cold probe was added. To further verify binding specificity, promoter probes carrying deletions in the predicted VipR-binding motifs were generated for *amidase* **(I)**, *cry2Ab* **(J)**, *cry1Ia* **(K)**, and *cry2Aa* **(L)** promoters. The positions of protein-DNA complexes (shifted bands) and free probe are indicated.

To evaluate whether VipR modulates the transcription of *cry* genes located within BtPAI-1, we analyzed the expression profiles of several *cry* genes in HD-1 and HD-1Δ*vipR* at different growth stages by using quantitative reverse transcription PCR (qRT-PCR). During the vegetative phase (9 h), transcription of *cry1Aa*, *cry2Ab*, *cry1Ia* and *cry2Aa* in HD-1Δ*vipR* was markedly reduced compared with HD-1, with relative expression levels of approximately 0.046-fold, 0.040-fold, 0.067-fold and 0.117-fold, respectively (Fig 2A–2D). Upon entry into the early sporulation phase (24 h), the expression of these genes in HD-1*ΔvipR* remained lower than in HD-1, showing 0.437-fold, 0.470-fold, 0.239-fold, and 0.610-fold of HD-1, respectively (Fig 2A–2D). By the late sporulation (36 h), transcription levels were similar in both strains with differences of less than 0.2-fold (Fig 2A–2D). These results indicate that in HD-1, VipR promotes transcription of multiple *cry* genes during vegetative growth and early sporulation, but has little or no effect on their expression during late sporulation.

To further investigate the role of VipR in regulating *cry* gene expression, we first examined transcriptional organization within the genomic region of BtPAI-1. RT-PCR analysis showed that each one of *cry2Ab*, *cry1Ia* and *cry2Aa* genes is transcribed independently, whereas *cry1Aa* is co-transcribed with the upstream *amidase* gene (S4 Fig). Based on these results, the promoter regions of *cry2Ab* (P_*cry2Ab*_), *cry1Ia* (P_*cry1Ia*_), and *cry2Aa* (P_*cry2Aa*_), as well as the amidase promoter (P_*amidase*_) (S5 Fig) were fused to a *lacZ* reporter gene. Each promoter-*lacZ* fusion construction was introduced separately into both HD-1 and HD-1Δ*vipR*, and β-galactosidase activity was determined throughout bacterial growth. Promoter activities driven by P_*amidase*_, P_*cry2Ab*_ and P_*cry1Ia*_ were significantly higher in HD-1 than in HD-1Δ*vipR* across all tested growth phases (Fig 2E–2H). Specifically, at 9 h (vegetative phase), P_*amidase*_-driven β-galactosidase activity in HD-1 reached approximately 100 units, whereas it was nearly undetectable (0 units) in HD-1Δ*vipR* (Fig 2E). At 12 h (vegetative phase), the P_*cry2Ab*_-driven β-galactosidase activity in HD-1 was 153 units, about seven-fold higher than that in HD-1Δ*vipR* (Fig 2F). Similarly, at 14 h (late vegetative phase), the P_*cry1Ia*_-driven activity in HD-1 reached 56 units, but remained nearly undetectable in HD-1Δ*vipR* (Fig 2G). In each case, peak promoter activity in HD-1 was 5.3-fold (P_*amidase*_), 3.8-fold (P_*cry2Ab*_) and 1.3-fold (P_*cry1Ia*_) higher than that in HD-1Δ*vipR*, respectively (Fig 2E–2G). In contrast, activity of the P_*cry2Aa*_ promoter showed a different temporal pattern. Before 28 h, the β-galactosidase activity driven by P_*cry2Aa*_ was significantly higher in HD-1 than in HD-1Δ*vipR*, with a peak activity of approximately 4.9-fold higher in the wild type strain. After 28 h, however, promoter activity became higher in HD-1Δ*vipR* than in HD-1 (Fig 2H). Taken together, these results indicate that in HD-1, VipR activates *cry* gene transcription during vegetative growth by modulating promoter activity.

To determine whether VipR directly regulates these promoters, we purified recombinant VipR protein and performed electrophoretic mobility shift assays (EMSA). DNA probes corresponding to P_*amidase*_, P_*cry2Ab*_, P_*cry1Ia*_ and P_*cry2Aa*_ all showed clear binding to VipR protein (Fig 2I–2L). These interactions were specific, as the shifted bands were efficiently competed away by addition of excess unlabeled competitor DNA probes (Fig 2I–2L). Deletion of the predicted VipR-binding motifs in the promoter regions of *amidase* (Fig 2I), *cry2Ab* (Fig 2J), *cry1Ia* (Fig 2K), and *cry2Aa* (Fig 2L) completely abolished VipR binding to these mutant probes, further supporting the sequence-specific interaction between VipR and these promoters. Collectively, these data demonstrate that VipR directly binds to the promoter regions of multiple cry genes within BtPAI-1 of HD-1 strain and activates their transcription during vegetative growth.

### Overexpression of *vipR* increases transcription of BtPAI-1 toxin genes during the vegetative phase and enhances insecticidal activity

Given that transcription factor-mediated regulation is often dose-dependent [36], we hypothesized that increasing the transcriptional level of *vipR* could result in enhanced transcription of *cry* genes, thereby improving the insecticidal activity of the modified strains. To test this hypothesis, *vipR* was placed under the control of the constitutive promoter P_*kan*_ and introduced into two Bt strains (HD-1 and CT-43), which harbor BtPAI-1. The qRT-PCR analysis confirmed that *vipR* transcript levels were significantly increased in all strains carrying the P_*kan*_-*vipR* construct, with increases of 43-fold in HD-1, and 74-fold in CT-43 relative to their respective wild-type controls (S6 Fig).

**Fig 3:**
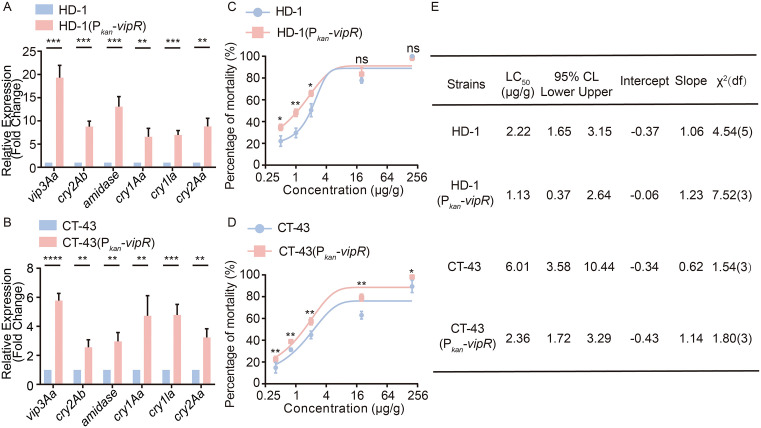
Overexpression of *vipR* significantly upregulates the transcriptional levels of multiple insecticidal protein genes in BtPAI-1 during the vegetative phase and enhances insecticidal activity. **(A, B)** The qRT-PCR analysis of *vip3Aa*, *cry2Ab*, *amidase*, *cry1Aa*, *cry1Ia* and *cry2Aa* transcriptional levels in HD-1, CT-43, and their respective *vipR* overexpression strains (HD-1 (P_*kan*_-*vipR*) and CT-43 (P_*kan*_-*vipR*)) at 9 h. The *gapdh* and *16S rRNA* genes were used as internal reference genes. Data represent the means ± SD of three biological replicates (n = 3). Statistical significance was determined using appropriate tests as explained in Materials and methods. Significance thresholds: ns, *P* > 0.05, *P < 0.05, **P < 0.01, ***P < 0.001, ****P < 0.0001. **(C, D)** Larval mortality of *Spodoptera frugiperda* following exposure to spore-crystal mixture samples derived from *vipR*-overexpressing strains and their corresponding wild-type strains. Spore-crystal mixtures were prepared as described in Fig 1E (60 h cultures, complete sporulation, adjusted to 1 × 10^7^ CFU/mL, lyophilized) and serially diluted to final concentrations of 1000, 100, 10, 5, 2.5, 1.25 and 0.625 μg/mL. The serially diluted spore-crystal suspensions were mixed with artificial diet for bioactivity assays. Data represent the means ± SD (n = 3 biological replicates). ns, *P* > 0.05, *P < 0.05, **P < 0.01, ***P < 0.001, ****P < 0.0001. **(E)** Toxicity assays of spore-crystal mixture from HD-1, CT-43, and their respective *vipR* overexpression strains (HD-1 (P_*kan*_-*vipR*) and CT-43 (P_*kan*_-*vipR*)) against *S. frugiperda* larvae. LC_50_ refers to the concentration causing 50% mortality of target insects, expressed as μg lyophilized spore-crystal mixture per gram of diet. LC_50_ values, along with their 95% confidence limits (95% CL), intercept, slope, and Chi-square (χ^2^) values with corresponding degrees of freedom (df), are presented in the figure.

We next assessed the transcriptional response of insecticidal genes encoded within BtPAI-1 during the vegetative phase (9 h). In HD-1, overexpression of *vipR* resulted in significant upregulation of *vip3Aa*, *cry2Ab*, *cry1Aa*, *cry1Ia* and *cry2Aa*, with significant fold changes of approximately 19.3-fold, 8.8-fold, 6.6-fold, 7.0-fold, and 8.8-fold, respectively (Fig 3A). In CT-43, the same genes were upregulated by approximately 5.8-fold, 2.6-fold, 4.7-fold, 4.8-fold and 3.2-fold, respectively (Fig 3B). These results indicate that increased *vipR* expression promotes the transcription of multiple toxin genes in the HD-1 and CT-43 strains.

We then assessed whether these transcriptional changes affected insecticidal activity. Spore-crystal mixtures from *vipR*-overexpressing and wild-type strains were tested against *S. frugiperda* larvae across a range of concentrations. At high concentrations, both HD-1 and its *vipR*-overexpressing derivative exhibited potent insecticidal activity, with no significant differences in larval mortality (Fig 3C). In contrast, at lower concentrations, the *vipR*-overexpressing strain of HD-1 consistently showed higher larval mortality than its corresponding wild-type strains (Fig 3C). For strain CT-43, *vipR* overexpression resulted in increased larval mortality relative to the wild type across all tested concentrations (Fig 3D). Consistent with these phenotypic trends, LC_50_ estimates indicated an approximately two-fold increase in insecticidal activity for the HD-1 *vipR*-overexpressing strain and a three-fold increase for the CT-43 counterpart compared with their respective wild-type controls (Fig 3E). However, it is important to mention that the overlap in the 95% confidence limits for HD-1 indicates that this difference was not statistically significant (Fig 3E). This discrepancy is likely attributable to the compensation in crystal production that is activated at the late stages of sporulation.

Collectively, these findings show that *vipR* overexpression significantly enhances the transcription of BtPAI-1-encoded *cry* genes in HD-1 and CT43, and this transcriptional upregulation contributes to a moderate improvement of insecticidal activity.

### The *vipR* is widely distributed among Bt strains and associated with BtPAI-1 and its variants

**Fig 4:**
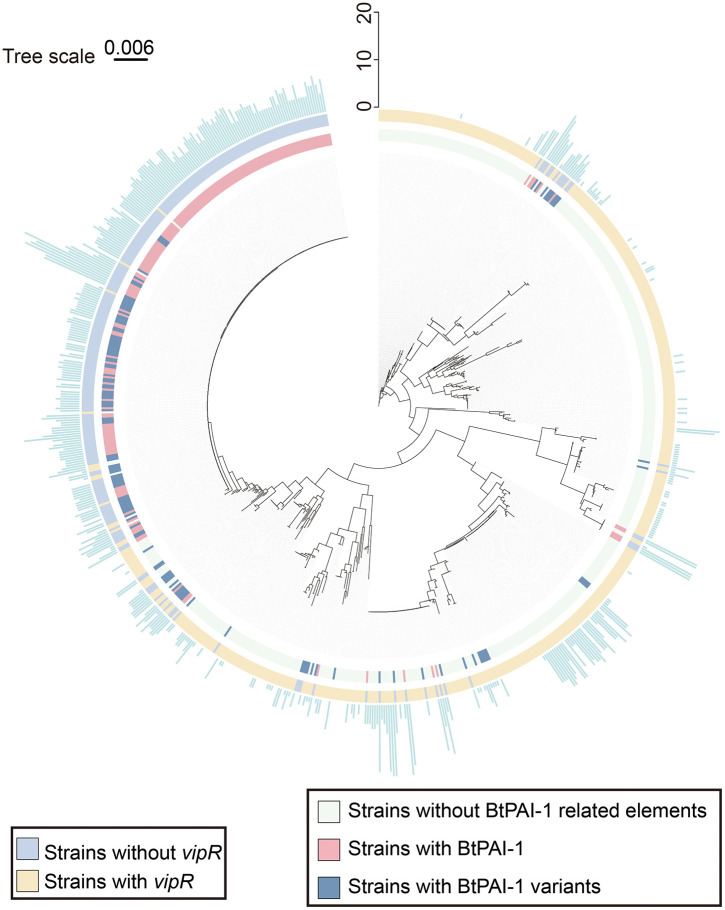
*vipR* is widely distributed in Bt strains and is associated with BtPAI-1 and its variants. The maximum likelihood (ML) phylogenetic tree of Bt strains was inferred using PhyML with the best-fit substitution model (JTT + I + G), based on alignments of concatenated protein sequences of single-copy core genes. Bootstrap support values were calculated from 1000 replicates. The outermost ring indicates the presence of *vipR* (light coral, *vipR*-positive; and light teal green, *vipR*-negative strains). The inner ring denotes the distribution of BtPAI-1 and its variants. The bar plot surrounding the tree represents the number of insecticidal protein genes identified in each Bt strain.

To assess the distribution of *vipR* and BtPAI-1 among Bt populations, we performed a systematic analysis in 889 publicly available Bt genomes from the NCBI database. BtPAI-1 or its homologous sequences (BtPAI-1 variants) were identified in 327 strains (37%). Among these, 196 strains contained a complete BtPAI-1, whereas 131 strains carried partial regions, hereafter referred to as BtPAI-1 variants (Figs 4 and S7).

We further analyzed the distribution of *vipR* across these Bt genomes. A total of 296 strains (33%) harbored *vipR* (Fig 4). The presence of *vipR* was strongly associated with BtPAI-1 or its variants (Fig 4). Among these *vipR*-positive strains, 196 strains (66%) contained *vipR* within complete BtPAI-1 elements, while the remaining 100 strains carried *vipR* located adjacent to BtPAI-1 variants (Fig 4). Conversely, only 24% of the strains harboring BtPAI-1 variants lacked *vipR* (Fig 4). Furthermore, all strains harboring *vipR* encoded more than five major insecticidal protein genes (Fig 4). These findings indicate that *vipR* is widely distributed in Bt, and is closely associated with BtPAI-1 and variants, suggesting that *vipR* as a conserved regulator of toxin gene expression.

### Conserved role of VipR among different Bt backgrounds

As mentioned above, 24% of analyzed Bt strains that harbor BtPAI-1 or its variants, lack a *vipR* gene. Bioinformatic analysis of these strains further revealed that the *cry* genes and their corresponding promoter regions within BtPAI-1 and its variants exhibit a high nucleotide identity (>80%), with putative VipR-binding sites within their promoter regions (S8 Fig). Based on these observations, we hypothesized that introducing *vipR* into these strains would trigger premature activation of insecticidal gene expression.

**Fig 5:**
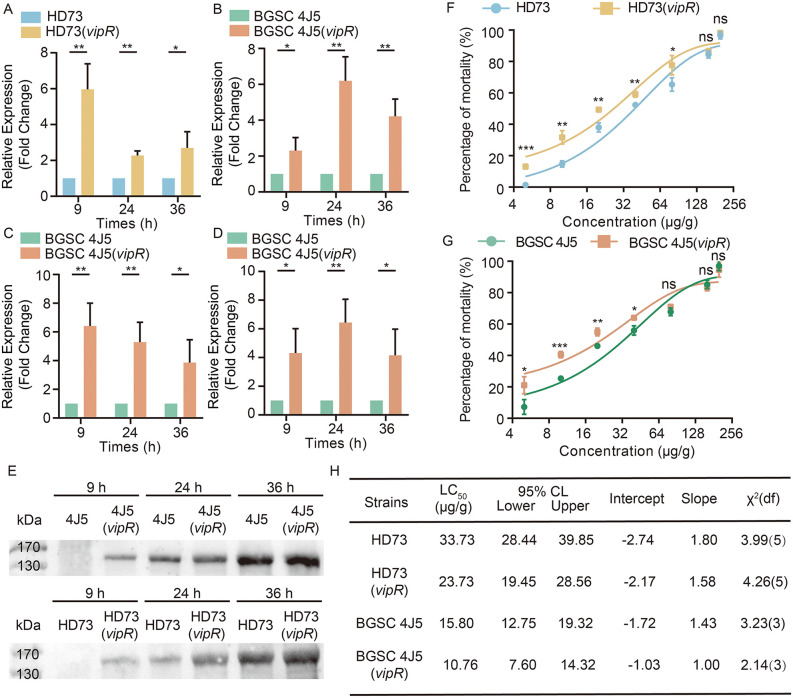
VipR promotes premature Cry proteins expression during the vegetative phase and enhances insecticidal activity in *vipR*-deficient Bt. **(A)** qRT-PCR analysis of *cry1Ac* transcript levels in wild-type HD73 and HD73 transformed with *vipR* gene, at 9 h, 24 h, and 36 h of growth. The *gapdh* and *16S rRNA* genes were used as internal reference genes. Data represent the means ± SD from three independent biological replicates (n = 3). Statistical significance was described in Materials and methods. Significance thresholds: ns, *P* > 0.05, *P < 0.05, **P < 0.01, ***P < 0.001, ****P < 0.0001. **(B–D)** qRT-PCR analysis of *cry2Ab, cry1Aa* and *cry1Ia* transcriptional levels in wild-type BGSC 4J5 and BGSC 4J5(*vipR*) transformed with *vipR* gene, at 9 h, 24 h, and 36 h of growth. The internal reference genes, data representation and statistical analyses are as described in Fig 5A. **(E)** Western blot analysis of Cry1A protein accumulation in HD73, BGSC 4J5, HD73 (*vipR*) and BGSC 4J5 (*vipR*) at 9 h, 24 h and 36 h. Sample preparation and loading were performed as described in Fig 1C (cells adjusted to 1 × 10^7^ CFU/mL; 20 µL per lane, equivalent to 2 × 10^5^ CFU per lane). Cry1A protein was detected using anti-Cry1A primary antibody and a goat anti-rabbit IRDye 680 secondary antibody. Data are representative of three independent experiments. **(F, G)** Larval mortality of *Spodoptera frugiperda* larvae after exposure to spore-crystal mixture samples collected from HD73, BGSC 4J5, HD73(*vipR*) and BGSC 4J5(*vipR*) cultures. Sample preparation was performed as described in Fig 1E (60 h cultures, complete sporulation, adjusted to 1 × 10^7^ CFU/mL, lyophilized) and serially diluted to final concentrations of 1000, 100, 10, 5, 2.5, 1.25, and 0.625 μg/mL. The serially diluted spore-crystal suspensions were mixed with artificial diet for bioactivity assays. Data represent the means ± SD (n = 3 biological replicates). ns, *P* > 0.05, *P < 0.05, **P < 0.01, ***P < 0.001, ****P < 0.0001. **(H)** Insecticidal activity of spore-crystal mixture from HD73, BGSC 4J5, HD73(*vipR*) and BGSC 4J5(*vipR*) against *S. frugiperda* larvae. LC_50_ values refer to the sample concentration causing 50% mortality of target insects, expressed as μg of lyophilized spore-crystal mixture per gram of diet. LC_50_ values, along with their 95% confidence limits (95% CL), intercept, slope, and Chi-square (χ²) values with corresponding degrees of freedom (df), are indicated in the figure.

To test this hypothesis, *vipR* was heterologously expressed in strains HD73 [37] and BGSC 4J5 [19], both of which lack an endogenous *vipR* gene. We then examined the transcriptional levels of *cry* genes within their respective BtPAI-1 variants and evaluated the insecticidal activity of the engineered strains. The qRT-PCR analysis revealed that *cry1Ac* transcription in HD73 (transformed with *vipR*) was significantly increased during the vegetative phase (9 h), exhibiting an approximately 6.0-fold increase relative to the parental HD73 strain (Fig 5A). Similarly, BGSC 4J5 (transformed with *vipR*) displayed increased transcript levels of *cry2Ab, cry1Aa* and *cry1Ia* transcripts at 9 h, with approximately 2.3-fold, 6.4-fold and 4.3-fold increases, respectively (Fig 5B–5D). Notably, the elevated transcription of these genes persisted in the *vipR*-expressing strains after entry into the stationary phase (24 h-36 h) compared to the wild-type strains (Fig 5A–5D). Consistent with the transcriptional data, western blot analysis demonstrated early accumulation of Cry proteins in the *vipR*-expressing strains. Cry1Ac was detected as early as 9 h in both HD73 (*vipR*) and BGSC 4J5 (*vipR*), whereas prominent Cry1A signals in the parental strains were only observed after the stationary phase (24 h and 36 h) (Fig 5E). Then, we performed bioassays using spore-crystal mixtures against the susceptible insect larvae. At low concentrations, strains expressing heterologous *vipR* gene exhibited higher larval mortality than the wild-type strain (Fig 5F and 5G). In contrast, at high concentrations, all strains displayed potent insecticidal activity, with no significant differences in larval mortality among groups (Fig 5F and 5G). Consistent with this trend, LC_50_ estimates indicated that the insecticidal activity of the *vipR*-expressing strains, HD73 (*vipR*) and BGSC 4J5 (*vipR*), increased by approximately two-thirds relative to the wild type. However, again the 95% confidence limits overlapped, indicating that these differences were not statistically significant (Fig 5H). Heterologous expression of *vipR* markedly enhanced the transcription of *cry* genes during the vegetative phase in Bt strains HD73 and BGSC 4J5 carrying BtPAI-1 variants. However, this elevated transcription did not translate into a statistically significant increase in the insecticidal activity of sporulated cultures. A plausible explanation, consistent with the overexpression scenario, is that compensatory crystal production during the late stages of sporulation offsets the potential advantages conferred by increased gene expression.

Collectively, these results demonstrate that heterologous expression of *vipR* is sufficient to induce vegetative-phase transcription of *cry* genes in the Bt strains HD73 and BGSC 4J5 carrying BtPAI-1 variants, leading to earlier accumulation of Cry proteins. Moreover, these findings also indicate that the regulatory function of VipR may be conserved across diverse Bt strains harboring distinct BtPAI-1 variants.

### VipR activates transcription of non-PAI insecticidal genes during the vegetative phase

**Fig 6:**
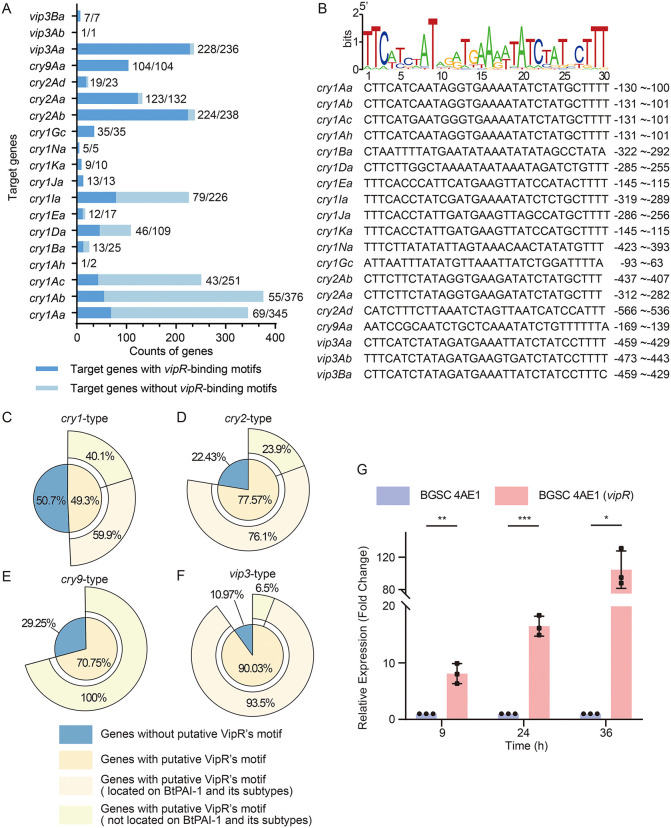
VipR activates the transcription of multiple potential target genes distributed across the Bt genome during the vegetative phase. **(A)** Identification of potential regulatory targets of VipR based on its conserved binding motifs. Proportion of genes harboring conserved VipR-binding motifs among homologous insecticidal protein gene families. **(B)** Predicted conserved VipR-binding motifs in the promoter regions of individual target genes. **(C–F)** Pie chart showing the percentage of *cry1*-type genes **(C)**, *cry2*-type genes **(D)**, *cry9*-type genes **(E)** and *vip3*-type genes **(F)** carrying VipR-binding motifs located on BtPAI-1 (and its variants) or in independent genomic regions. **(G)** Detection of *cry9Aa* transcriptional levels in BGSC 4AE1 and BGSC 4AE1(*vipR*). The qRT-PCR analysis of *cry9Aa* in BGSC 4AE1 and BGSC 4AE1 (*vipR*) was performed at 9 h, 24 h and 36 h. The *gapdh* and *16S rRNA* genes were used as internal reference genes. Data represent the means ± SD (n = 3 biological replicates). Statistical significance was determined using appropriate tests (see Materials and methods). Significance thresholds: *P < 0.05, **P < 0.01, ***P < 0.001.

We have demonstrated that VipR directly binds to the promoter regions of different BtPAI-1-associated insecticidal genes and activates their expression in multiple Bt strains. Our EMSA assays confirmed that deletion of the predicted VipR-binding motif abolishes DNA binding, identifying this sequence as a key determinant site for VipR-promoter interaction (Fig 2I–2L). Furthermore, similar motifs have been identified in the promoter region of additional insecticidal genes, including *cry9Ca-orf1* and *cry9Aa* genes [38], suggesting that VipR may regulate a broader set of targets (Fig 6A and 6B). Consistent with this possibility, our systematic genome-wide bioinformatic analysis performed on 889 available Bt genomes, where insecticidal protein genes were identified using BtToxin_Digger [39], and their promoter regions were scanned for the potential VipR-binding motif using the FIMO tool (MEME Suite v5.5.4, http://meme-suite.org/tools/fimo) [40], revealed that potential VipR-binding motifs are widely distributed among different classes of Bt insecticidal genes: detected in 49.32% of *cry1*-type genes (831/1685), 77.57% of *cry2*-type genes (377/486), 70.75% of *cry9*-type genes (104/147), and 90.3% of *vip3*-type genes (307/341) (Fig 6C–6F). We also found that 59.9% of *cry1*-type, 76.1% of *cry2*-type, and 93.5% of *vip3*-type genes are located on BtPAI-1 or its variants (Fig 6C–6F). In contrast, 40.1% of *cry1*-type genes, 23.9% of *cry2*-type genes, 6.5% of *vip3*-type genes and 100% of *cry9*-type genes are located on non-BtPAI-1 plasmids (Fig 6C–6F). Furthermore, when we introduced *vipR* into the *cry9Aa*-harboring strain BGSC 4AE1, we observed a significant increase in the transcription level of the *cry9Aa* gene (Fig 6G), suggesting that VipR may regulate both BtPAI-1-encoded and non-BtPAI-1-encoded insecticidal genes across diverse Bt genetic backgrounds such as HD-1, CT-43, HD73, BGSC 4J5 and BGSC 4AE1 strains.

## Discussion

Pathogenicity islands (PAIs) are mobile genetic elements that play a central role in bacterial virulence [10]. Beyond serving as repositories for virulence genes, PAIs frequently encode regulatory components that coordinate the spatiotemporal expression of these genes [6]. In Bt, the insecticidal protein genes are predominantly clustered within plasmid-borne PAIs [19], and their expression has long been considered strictly dependent on the σ^E^/σ^K^ regulatory cascade during sporulation, establishing a paradigm of sporulation-dependent toxin production [20,21]. However, previous studies have shown that engineering earlier expression of insecticidal genes via promoter replacement can significantly enhance the toxicity of Bt strains [30,31], and that transcriptional regulators such as CpcR can activate insecticidal gene expression during the vegetative phase in asporogenous strains [32,33]. These observations suggest that endogenous regulatory mechanisms may enable Bt to control insecticidal gene expression prior to sporulation. In this study, we identified VipR, an endogenous transcriptional regulator encoded within BtPAI-1, which directly activates the transcription of different BtPAI-1 insecticidal genes (*cry1Aa*, *cry1Ia*, *cry2Ab*, *cry2Aa*) during the vegetative phase in HD-1 and CT-43 strains. This premature activation correlates with increased accumulation of insecticidal Cry proteins and enhanced insecticidal activity. We further demonstrate that *vipR* is widely distributed among Bt lineages and is consistently associated with BtPAI-1 or its variants. Furthermore, heterologous expression of *vipR* in Bt strains HD73 and BGSC 4J5 lacking this gene but harboring BtPAI-1 variants is sufficient to trigger vegetative-phase expression of *cry* genes and promote early Cry protein accumulation. Together, these findings demonstrated that BtPAI-1 itself, encodes an intrinsic regulatory module that reprograms the timing of insecticidal gene expression independently of the sporulation-specific σ-factor cascade.

Although *vipR* is strongly associated with BtPAI-1 and its variants, approximately 24% of BtPAI-1 variants lack this gene (S7 Fig), indicating a dynamic evolutionary history. One possibility is a “gene loss” trajectory, analogous to the progressive reduction of virulence-associated genes observed in other pathogenicity islands, such as SPI-1 in *Salmonella* [41]. Alternatively, *vipR* may have been acquired through horizontal gene transfer and selectively retained due to the advantage conferred by premature activation of insecticidal gene expression [42]. This scenario parallels the acquisition and stable maintenance of key regulatory factors, such as *enrR* in *Edwardsiella piscicida*, which enhance virulence and fitness [43]. Our functional data provide strong support for the latter model. We show that introduction of *vipR* into BtPAI-1 variants strains lacking this regulator such as HD-73 and BGSC 4J5, was sufficient to restore high-level vegetative-phase expression of insecticidal proteins and early Cry protein accumulation (Fig 5A–5E). These findings indicate that *vipR* functions as an independent and transferable regulatory module and reinforce the concept that BtPAIs encode not only clusters of virulence and insecticidal genes, but also the regulatory circuits required for their coordinated expression.

**Fig 7:**
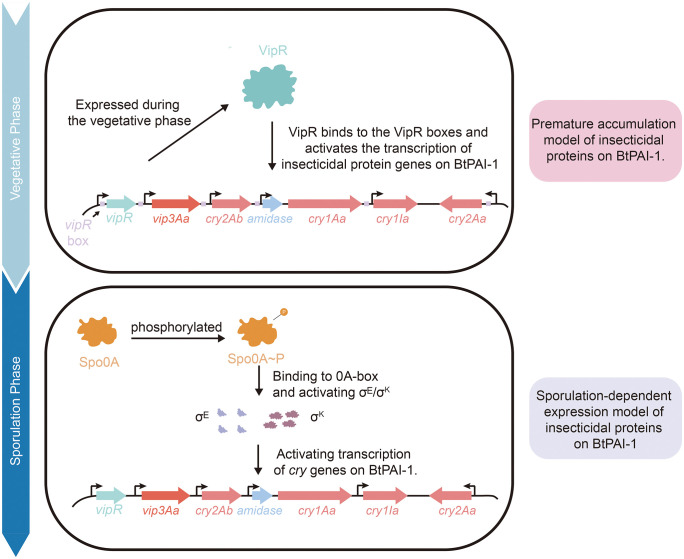
Two-stage coordinated regulatory model for BtPAI-1-associated insecticidal gene expression in Bt. During the vegetative phase, VipR is expressed and directly binds to its target boxes in promoter regions within BtPAI-1, thereby prematurely activating the transcription of multiple insecticidal protein genes and promoting premature Cry protein accumulation. Upon entry into sporulation, phosphorylated Spo0A (Spo0A~P) initiates the sporulation program by activating the σ^E^/σ^K^ regulatory cascade. These σ factors, together with RNA polymerase, drive high level transcription of *cry* genes through recognition of sporulation-specific promoter elements. This two-stage regulatory model ensures both early and sustained production of insecticidal proteins, thereby enhancing the insecticidal activity of the bacterium.

Our results further demonstrate that the regulatory activity of VipR is temporally restricted. Deletion of *vipR* markedly reduced the transcription and protein accumulation of BtPAI-1-associated insecticidal genes during the vegetative phase (9 h) and early sporulation phase (24 h), whereas its effect was minimal during late sporulation (36 h) (Fig 2A–2H). These observations indicate that the regulatory function of VipR is predominantly restricted to the vegetative and early sporulation phases, and the expression of insecticidal protein genes during late sporulation is likely regulated by other factors. Previous studies have confirmed that the promoter regions of BtPAI-1 insecticidal genes, such as *cry2Ab*, *cry1Aa*, *cry1Ia*, contain σ^E^/σ^K^ binding motifs likely involved in the expression of insecticidal genes during sporulation [21]. Therefore, we propose a two-stage coordinated regulatory model for BtPAI-1-associated insecticidal gene expression. In this model, VipR directly binds to target promoters and activates insecticidal protein gene transcription, enabling premature accumulation of insecticidal proteins during vegetative phase (Fig 7). Upon entry into sporulation, activation of the σ^E^/σ^K^ regulatory cascade replaces VipR-dependent control and becomes dominant, driving robust transcription of insecticidal genes during sporulation, ensuring sustained high-level expression and formation of spore–crystal complexes. (Fig 7).

Independent support for this model is provided by a recent preprint work from Verplaetse et al. (2025), who showed that VipR promotes premature expression of Cry proteins during the vegetative phase [44]. By introducing VipR into HD73 and HD-1 strains and analyzing a *spo0A* deletion background, they showed that VipR-mediated activation of Cry protein production occurs independently of the sporulation cascade. Our study extends these findings by providing direct mechanistic evidence of VipR-dependent transcriptional activation, including the demonstration of specific VipR-DNA interactions through EMSA assays and the quantitative analysis of gene expression dynamics across growth phases (Fig 2). Together, these complementary approaches establish VipR-mediated vegetative-phase expression as a key regulatory strategy controlling the timing of insecticidal protein production in Bt.

Interestingly, we show that *vipR* transcription persists during late sporulation (S9 Fig), despite the limited effect of its deletion showed slight effect on insecticidal gene expression at this growth phase. This result suggests that VipR is not a major regulator during late sporulation and raises the possibility that it may have additional uncharacterized biological functions, beyond the control of BtPAI-1-associated insecticidal genes. Further studies will be required to elucidate these potential roles.

Our findings further demonstrate that insecticidal protein expression in Bt is not restricted to the sporulation phase. Previous studies reported that the transcriptional regulator CpcR in strain LM1212 can activate Cry protein expression during vegetative growth. However, this activity is restricted to sporulation-deficient strains and does not occur in strains capable of normal sporulation [33]. In contrast, our results demonstrate that approximately 33% (296/889) of Bt strains encode the transcriptional regulator VipR (Fig 4), which directly activates the transcription of multiple *cry* genes during the vegetative phase (Fig 2). This widespread distribution together with its direct regulatory activity, enables premature Cry protein accumulation and enhances insecticidal activity across diverse Bt genetic backgrounds. Upon entry into sporulation, additional regulatory factors, including the σ^E^/σ^K^ cascade, mediate sustained high-level transcription of *cry* genes, ultimately leading to the formation of spore-crystal mixtures. These findings support a conserved two-stage expression regulation strategy, characterized by VipR-dependent vegetative phase activation followed by sporulation-dependent expression (Fig 7). This coordinated mechanism maximizes insecticidal protein accumulation while ensuring the production of stress-resistant spores, thereby contributing to the environmental persistence and stability of Bt-based formulations. It is proposed that early expression of Cry toxins induced by VipR may have a fundamental role *in vivo* expression of toxin genes during infection of Bt spores in the absence of insecticidal crystals, supporting the evolutionary role of the VipR transcription regulator. Further studies are required in this aspect.

In addition to its biological significance, VipR has important applied implications. We show that overexpression of *vipR* or heterologous introduction of *vipR* into different Bt strains (HD-1, CT-43, HD-73, BGSC 4J5 and BGSC 4AE1) advances the timing of insecticidal gene expression (*cry1Aa*, *cry1Ac*, *cry1Ia*, *cry2Aa*, *cry2Ab* and *cry9Aa*) and enhances their insecticidal activity. These results suggest that *vipR* can serve as a molecular marker for the identification of highly-virulent Bt strains, enabling more efficient screening of isolates with enhanced insecticidal potential. Moreover, modulating *vipR* expression either through upregulation or genetic introduction could represent a promising strategy for improving the efficacy of Bt-based pesticides.

In conclusion, we identify VipR as a BtPAI-1-encoded transcriptional regulator that directly binds to promoter regions of insecticidal genes and activates their expression during the vegetative phase, thereby promoting premature Cry protein accumulation. These findings revise the current paradigm that *cry* gene expression is strictly restricted to sporulation and establish a two-stage regulatory model in which VipR-mediated early activation is followed by σ^E^/σ^K^-dependent expression during sporulation. Furthermore, our results demonstrate that manipulation of *vipR* expression enhances insecticidal protein production, providing a rational framework for the screening and genetic engineering of highly virulent Bt strains for biopesticide development.

## Materials and methods

### Bacterial strains, plasmids, and cultural conditions

*Escherichia coli* DH5α was used as the host strain for plasmid construction and *E. coli* BL21(DE3) [45] was used for VipR protein production. All bacterial strains and plasmids used in this study are listed in S1 and S2 Tables, respectively. *E. coli* strains were cultured in Luria-Bertani (LB) medium (0.5% yeast extract, 1% NaCl, 1% peptone) [46] 37 °C with shaking at 220 rpm. *Bacillus thuringiensis* (Bt) was cultured in insecticidal crystal protein medium (ICPM: 0.6% peptone, 0.5% glucose, 0.05% MgSO_4_·7H_2_O, 0.1% CaCO_3_, 0.05% KH_2_PO_4_; pH 7.0) at 28 °C with shaking at 220 rpm. Antibiotics were used at the final concentrations: erythromycin (10 μg/mL), kanamycin (100 μg/mL), ampicillin (100 μg/mL), and spectinomycin (100 μg/mL).

### DNA manipulation

Standard molecular biology procedures were performed according to Sambrook and Russell, 1983 [47]. DNA fragments from gels or PCR products were purified using the E.Z.N.A. Gel Extraction Kit (Omega). Primers used in this study are listed in S3 Table. All recombinant plasmids were verified by Sanger sequencing (Augct, Wuhan, China). *E. coli* was transformed using the standard chemical method [47], while Bt was transformed by electroporation as previously described [48].

### Phylogenetic analysis

Protein sequences from Bt genomes were subjected to BlastP analysis (E-value < 10^−5^), followed by clustering analysis using the Markov cluster algorithm (MCL) with an inflation parameter of 2 [49]. Single-copy core proteins from each cluster were aligned using MUSCLE [50], and poorly aligned regions were trimmed with TrimaAl [51]. A maximum likelihood (ML) phylogenetic tree was constructed using PhyML with the best-fit substitution model (JTT + I + G).

Genome assemblies were retrieved from GenBank, and those with scaffold N50 < 50 kb were excluded. Core genome single nucleotide polymorphisms (SNPs) were identified using the Northern Arizona SNP Pipeline (NASP) (https://github.com/TGenNorth/NASP).

To determine the distribution of BtPAI-1 and its variants, BLASTP searches (E-value <1 x 10^-5^) were performed for VipR, Vip3Aa, Cry2Ab, amidase, Cry1Aa, and Cry1Ia across all Bt genomes. The positional relationships of these genes were analyzed, and the phylogenetic tree was annotated using iTOL [52].

### CRISPR-Cas9 mediated gene deletion

The *vipR* and *vip3Aa* gene in Bt was knocked out using the CRISPR-Cas9 system based on plasmid pJOE8999, as previously described [53]. Single guide RNA (sgRNA) targeting *vipR* and *vip3Aa* were designed using CRISPy-web (https://crispy.secondarymetabolites.org/#/input) [54] (see S3 Table for details). The corresponding sgRNA cassettes were generated using primer pairs del*vipR*-sgRNA-F/R and del*vip3Aa*-sgRNA-F/R and cloned into *Eco*31I-digested pJOE8999, respectively. For *vipR*, the upstream (507 bp) and downstream (422 bp) homologous arms of *vipR* gene were amplified using the delvipR-*sfi*I-up-F/R and delvipR-*sfi*I-down-F/R primers, respectively. For *vip3Aa*, the upstream (497 bp) and downstream (500 bp) homologous arms of *vip3Aa* gene were amplified using the del*vip3Aa*-*sfi*I-up-F/R and del*vip3Aa*-*sfi*I-down-F/R primers, respectively. These fragments were amplified and fused by using splicing by overlap extension (SOE) PCR technology. For each target gene, the homologous arms were fused by splicing by overlap extension (SOE)-PCR and inserted into *Sfi*I-digested pJOE8999, generating plasmids *vipR*-pJOE8999 and *vip3Aa*-pJOE8999, respectively. Each construct contained both the sgRNA cassette and the corresponding repair template. These construct were transformed into the HD-1 strain by electroporation to obtain the *vipR*-pJOE8999/HD-1 and *vip3Aa*-pJOE8999/HD-1 strains, respectively.

Transformants were cultured in LB medium at 28 °C with shaking at 220 rpm for 24 h. Next, 1 mL of the bacterial culture was diluted 10^4^–10^6^ times in LB medium, and 100 μL of this diluted culture was spread on LB solid medium. After overnight incubation at 28 °C, single colonies were picked from the plate and cultured overnight at 28 °C for further verification. Individual colonies were screened by colony PCR. Deletion of *vipR* was verified using primer pairs del*vipR*-upup-F/R and del*vipR*-dodo-F/R, whereas deletion of *vip3Aa* was verified using primer pairs del*vip3Aa*-upup-F/R and del*vip3Aa*-dodo-F/R. The resulting mutants were further confirmed by Sanger sequencing (S1 and S3 Figs).

To cure the temperature-sensitive pJOE8999 plasmid [55], the deletion mutant strain Δ*vipR*-pJOE8999/HD-1 and Δ*vip3Aa*-pJOE8999/HD-1 were inoculated into LB medium and cultured at 42 °C with shaking at 220 rpm for 24–48 h. Elimination of the pJOE8999 plasmid was confirmed by antibiotic sensitivity screening and PCR using primers pJOE8999-F/R.and

Deletion of genes and absence of off-target mutations were confirmed by Sanger sequencing of the mutated region. Sequencing reads were aligned to the reference genome of the wild-type HD-1 strain using SnapGene software with default parameters, and visualization was performed using Jalview to confirm the precise deletion. No off-target mutations were detected, confirming the accuracy of the knockout process.

### Bacterial growth curves

The wild-type HD-1 and the mutant deletion HD-1*ΔvipR* strains were cultured overnight at 28 °C in LB medium and subsequently diluted 1:100 into fresh LB medium. Cultures were grown to OD_600_ value of 0.4-0.5 at 28 °C with shaking at 220 rpm. Next, 200 μL aliquots were transferred to a 100-well honeycomb plate. Growth was monitored using a Bioscreen C Pro automated growth analyzer at 28°C with shaking at 480 rpm, recording OD_600_ every 30-min. All experiments were performed in triplicate.

### Insect bioassay

Strains HD-1 [56], CT-43 [57], HD-1Δ*vipR*, HD-1Δ*vip3Aa*, HD-1(P_*kan*_-*vipR*), CT-43(P_*kan*_-*vipR*), HD73, BGSC 4J5, HD73(*vipR*) and BGSC 4J5(*vipR*) were cultured in LB medium. Samples of fermentation broths of these strains were collected at the vegetative (9 h), the early sporulation (24 h) and late the sporulation (36 h) phases. Cell density of each sample was determined using the dilution plate counting method. Briefly, serial dilutions were prepared and plated onto LB agar plates, and colony-forming units (CFU) were calculated after incubation. Based on CFU determination, all samples were adjusted to a uniform density of 1 × 10^7^ CFU/mL using sterile double-distilled water (ddH_2_O). For bioassays, the standardized fermentation broth was further diluted four-fold (2.5 × 10^6^ CFU/mL). Subsequently, 3 mL of each diluted sample was mixed with 15 g of artificial diet, resulting in a final concentration of approximately 5 × 10^5^ CFU per gram of diet. The mixture was then aliquoted into 24-well plates for insecticidal activity assays (one larva per well).

In addition, for crystal-spore preparations, strains were cultured in ICPM medium at 28 °C with shaking at 220 rpm for 60 h (until complete sporulation, confirmed by microscopy). Cells were harvested by centrifugation (10,000 rpm, 4°C), washed 3–5 times with EDTA-NaCl solution (10 mM Ethylenediaminetetraacetic acid, 1M NaCl, pH 7.0), followed by three-times wash with ddH_2_O. The cell density of the washed sample was determined using the dilution plate counting method, and all samples were adjusted to a uniform density (1 × 10^7^ CFU/mL) with sterile ddH_2_O. The normalized samples were then lyophilized using a freeze dryer to obtain spore-crystal powders (Life Technologies). The lyophilized preparations were resuspended at 1000 μg/mL and serially diluted to final concentrations of 100, 10, 5, 2.5, 1.25 and 0.625 μg/mL. These suspensions were mixed with artificial diet for insect bioassays and LC_50_ values determinations.

3 mL of each diluted sample was thoroughly mixed with 15 g of artificial diet [58]. The mixed diet was transferred to 24-well culture plates, with one newly hatched neonate *S. frugiperda* larva per well. Each sample was performed in triplicate, and mortality was recorded after seven days. The LC_50_ values were calculated using probit analysis [59].

### Microscopy observation

For general observation, sporulated cultures were stained with fuchsin and observed under an optical microscope (Olympus, USA) using 100 x oil immersion objective.

### Western blot analysis

The Bt strains (HD-1, HD-1Δ*vipR*, HD73, HD73(*vipR*), BGSC 4J5, and BGSC 4J5(*vipR*)) were inoculated into 100 mL of LB medium and cultured at 28 °C with shaking at 220 rpm. Samples were collected at different growth phases (9 h, 24 h, and 36 h). For each sample, 2 mL of fermentation broth was centrifuged at 12,000 rpm for 2 min to collect the cell pellets. The pellets were washed twice with sterile ddH_2_O. The cell density of each sample was determined using the dilution plate counting method. Based on the results, all samples were adjusted to an equal cell density of 1 × 10^7^ CFU/mL with sterile ddH_2_O. Twenty microliters of each normalized sample was mixed with 5 µL of protein loading buffer, and the mixture was loaded per lane (equivalent to 2 × 10^5^ CFU per lane) according to the designated lane order.

Protein samples were separated by 10% SDS-polyacrylamide gel electrophoresis (SDS-PAGE) and transferred to a nitrocellulose (NC) membrane (Abclonal Technology, Wuhan, China) at 100 V for 90 min. Membranes were washed three times (5 min each) with PBST (137 mM NaCl, 2.7 mM KCl, 10 mM Na_2_HPO_4_, 1.8 mM KH_2_PO_4_, 0.05%(v/v) Tween-20, pH7.5), and blocked 2 h at room temperature with 5% bovine serum albumin, fraction Ⅴ dissolved in PBST (Colaber, Beijing, China). After washing three times with PBST (10 min each), the membranes were incubated 90 min with 1/5,000 dilution in PBST of anti-Cry1A antibody (provided by the Institute of Plant Protection, Chinese Academy of Agricultural Sciences). Following another three washes with PBST (10 min each), the membranes were incubated 45 min in the dark with IRDye 680-conjugated goat anti-rabbit secondary antibody (1/5,000 dilution in PBST) at room temperature. The membranes were washed three times with PBST (15 min each), and signals were finally acquired using a chemiluminescence imaging system (Tanon).

### RNA extraction and transcriptional analysis

The wild-type HD-1, the deletion mutant HD-1Δ*vipR* and additional strains including CT-43, HD-1(P_*kan*_-*vipR*), CT-43(P_*kan*_-*vipR*), HD73, BGSC 4J5, HD73(*vipR*) and BGSC 4J5(*vipR*) were inoculated into LB medium. Bacterial cultures were collected at the vegetative (9 h), late stationary (24 h), and sporulation (36 h) growth phases, by centrifugation (12,000 rpm, 4 °C). Total RNA was extracted by using the TransZol Up Plus RNA Kit (TransGen) and quantified with a NanoDrop ND-1000 spectrophotometer (Labtech, Wilmington, MA, USA). The cDNA synthesis was performed using PrimeScript RT reagent Kit with gDNA Eraser (Takara, Dalian, China). RT-PCR were conducted to detect the distribution of transcription units on the BtPAI-1, using RT-PCR primers listed in S3 Table. Quantitative reverse transcription PCR (qRT-PCR) assays were conducted using SYBR Green PCR Master Mix (Life Technologies, CA, USA) in an Applied Biosystems 7300 System (Applied Biosystems, Foster City, CA, USA). The gene expression levels were normalized using the *gapdh* and *16S rRNA* genes as an internal reference. All qRT-PCR primers are listed in S3 Table.

### Expression and purification of VipR

The codon optimized synthetic *vipR* gene was cloned into pET15D [60] (previously digested with *Nde*I and *Xho*I) via homologous recombination using the ClonExpress II One Step Cloning Kit (Vazyme). Positive clones were selected after verification by Sanger sequencing. The recombinant vector and the empty pET15D vector extracted from *E. coli* DH5α were introduced into *E. coli* BL21 (DE3) by chemical transformation. Colonies were screened by their ampicillin resistance and recombinant strains were identified by colony PCR.

The expression strain pET15D-*vipR*/BL21 was inoculated into LB medium supplemented with ampicillin and cultured at 37°C until the exponential phase (OD_600_ = 0.6-0.8). Subsequently, it was induced with 1 mM isopropyl-β-D-thiogalactopyranoside (IPTG) at 16 °C for 15 h. Cells were collected and homogenized in 40 mL lysis buffer (137 mM NaCl, 2.7 mM KCl, 10 mM Na_2_HPO_4_, 1.8 mM KH_2_PO_4_, 10 mM imidazole; pH7.5) on ice using a low-temperature ultra-high pressure continuous cell disruptor (JN-MiniPro). The cell lysate was clarified by centrifugation (60 min, 10,000 × *g* at 4 °C). The supernatant containing soluble proteins was applied to a Ni^2+^-nitrilotriacetate (Ni-NTA) affinity column (Qiagen) pre-equilibrated with lysis buffer. Nonspecifically bound proteins were removed with wash buffer (187 mM NaCl, 2.7 mM KCl, 10 mM Na_2_HPO_4_, 1.8 mM KH_2_PO_4_, 80 mM imidazole; pH7.5), followed by elution of the target protein with elution buffer (187 mM NaCl, 2.7 mM KCl, 10 mM Na_2_HPO_4_, 1.8 mM KH_2_PO_4_, 300 mM imidazole; pH7.5). Protein purity was verified by SDS-PAGE and buffer-exchange into 10 mM PBS buffer (137 mM NaCl, 2.7 mM KCl, 10 mM Na_2_HPO_4_, 1.8 mM KH_2_PO_4_) was performed by using a HiTrap Desalting Column and stored at -80 °C until used.

### Quantification of protein by SDS-PAGE and densitometry

The concentration of VipR was determined using SDS-PAGE combined with grayscale-based quantification using a BSA standard curve. Briefly, 40 µL of the protein sample was mixed with 10 µL of 5 × loading buffer and denatured by boiling for 10 min. For the standard curve, bovine serum albumin (BSA) was serially diluted to prepare protein standards at concentrations of 1000, 500, 250, 100, 50, 25, 12.5, 6.25, and 3.125 ng per lane. Both the VipR sample and BSA standards were resolved on a 10% SDS-polyacrylamide gel. After electrophoresis, the gel was stained with Coomassie Brilliant Blue R-250 and destained until clear background. The band intensities of both the BSA standards and the VipR protein were quantified using ImageJ software. A standard curve was generated by plotting the integrated density of BSA bands against their respective loading amounts, and the protein concentration of the VipR sample was calculated by interpolation.

### Electrophoretic mobility shift assays (EMSA)

The expression and purification of *vipR*-His protein were done as described above. The -10 and -35 promoter boxes of each candidate promoter was predicted using the Berkeley Drosophila Genome Project (BDGP) neural network promoter prediction tool [61]. The promoter DNA fragments were PCR amplified from Bt genomic DNA using specific primers labeled with a 5′-end FAM modification and confirmed by gel electrophoresis. EMSA assays were done as follows: In a 20 μL reaction system, the fluorescent labeled P_*vip3Aa*_, P_*cry2Ab*_, P_*amidase*_, and P_*cry1Ia*_ promoter fragments probes were incubated with purified VipR protein at different concentrations in binding buffer (10 mM Tris-HCl, 0.5 mM dithiothreitol (DTT), 50 mM NaCl, pH7.5, 4% (v/v) glycerol) at 37°C for 30 min. In addition, to exclude non-specific binding, 100 ng of poly(dI-dC) was added to each reaction mixture. For competition assays, a 10-fold molar excess of unlabeled specific cold probe was added. To further verify binding specificity, promoter probes carrying deletions in the predicted VipR-binding motifs were generated for *amidase*, *cry2Ab*, *cry1Ia*, and *cry2Aa* promoters and used as labeled probes in parallel reactions. The DNA-protein mixtures were applied to non-denaturing 5% (w/v) polyacrylamide gel in TBE buffer (90 mM Tris base, 90 mM boric acid, 2 mM EDTA, pH8.0) and electrophoresed at 120 V for 90 min. The labeled fragments were visualized using a Typhoon Scanner System (GE Healthcare).

### β-galactosidase activity assay

The promoter regions of *vip3Aa* (P_*vip3Aa*_, 413 bp), *cry2Ab* (P_*cry2Ab*_, 455 bp), *amidase* (P_*amidase*_, 347 bp), and *cry1Ia* (P_*cry1Ia*_, 457 bp) genes were amplified from HD-1 genomic DNA using Lacz-vip3Aa-F/R, Lacz-cry2Ab-F/R, Lacz-ami-F/R, and Lacz-cry1Ia-F/R primers, respectively (S3 Table). The P_*vip3Aa*_, P_*cry2Ab*_, P_*amidase*_, and P_*cry1Ia*_ fragments were digested with *Pst*I and *BamH*I, and ligated into the linearized pHT304-18z [62,63] plasmid which contains a promoter-less *lacZ* gene [58], to obtain the recombinant plasmids pHT304-P_*vip3Aa*_-lacZ, pHT304-P_*cry2Ab*_-lacZ, pHT304-P_*amidase*_-lacZ, and pHT304-P_*cry1Ia*_-lacZ. These recombinant plasmids were introduced into HD-1 and HD-1Δ*vipR*, respectively, to obtain recombinant strains HD-1(P_*cry2Ab*_-lacZ), HD-1(P_*vip3Aa*_-lacZ), HD-1(P_*amidase*_-lacZ), HD-1(P_*cry1Ia*_-lacZ), HD-1Δ*vipR*(P_*cry2Ab*_-lacZ), HD-1Δ*vipR*(P_*vip3Aa*_-lacZ), HD-1Δ*vipR*(P_*amidase*_-lacZ), and HD-1Δ*vipR*(P_*cry1Ia*_-lacZ). The successful construction of each strain was verified by PCR.

The β-galactosidase activity was measured in 2 ml samples collected by centrifugation (12,000 × g, 1min) from 100 ml cultures that were grown from 0 h to 36 h in LB medium. Pellets were stored at -20 °C util used. For the assay, the samples were resuspended in 0.5 mL of Z-buffer (0.06 M Na_2_HPO_4_, 0.04 M NaH_2_PO_4_, 0.01 M KCL, 1 mM MgSO_4_, 1 mM dithiothreitol). Cells were disrupted with glass beads (0.1 mm; BioSpec, USA) on ice using a JXFSTPRP-24 automatic rapid sample grinder (Jingxin, Shanghai, China). After centrifugation, the cell extracts were collected. Finally, 0.7 mL of Z-buffer supplemented with 0.2 mL of 4 mg mL-1 2-nitrophenyl β-D-galactopyranoside (sigma) were added to 100 µL of cell extract (ONPG; Sigma). The mixture was incubated at 37 °C and stopped by adding 0.5 mL of 1 M Na_2_CO_3_. Subsequently, the optical density of the reaction mixture was measured at 420 nm for the β-galactosidase assay. The protein concentration in the samples was determined using a Bio-Rad Protein Assay Kit with bovine serum albumin (BSA) as the standard. The specific activity of β-galactosidase was expressed as enzymatic activity units per milligram of protein (Miller units). These β-galactosidase activities were calculated using the formula: Unit value = (OD_420_ × 1500)/ (T × V × Protein concentration). Where T, is the reaction time (min) and V, is the volume of the sample added (μL). All data represent the mean ± standard error (SE) of at least three independent experiments.

### Construction of a strain overexpressing the *vipR* gene from Bt HD-1

The *vipR* overexpression vector was constructed using pHT315-*gfp* [62] plasmid, which contains the constitutive promoter P_*kan*_ and green fluorescent protein gene (*gfp*) under its control. The *gfp* gene was excised with the restriction endonucleases *Xba*I and *Hind*III, and ligated it with the *vipR* gene product, digested with the same restriction enzymes, to construct the recombinant plasmid pHT315-P_*kan*_-*vipR*. Subsequently, pHT315-P_*kan*_-*vipR* and pHT315-*gfp* were introduced into Bt HD-1 via electroporation to construct the *vipR* overexpression strain and the control Bt strains.

### Statistical analysis

Statistical analyses were performed using GraphPad Prism version 8.0. Data were compared between two groups, with detailed sample sizes. For normally distributed data, Levene’s test was first used to assess the homogeneity of variances. On the basis of outcome, either the independent samples t test (for equal variances) or Welch’s t test (for unequal variances) was applied. For non-parametric distributed data, the Mann-Whitney U test was used.

Statistical significance thresholds were set as follows: ns, *P* > 0.05; *P < 0.05; **P < 0.01; ***P < 0.001.

## Supporting information

S1 Table Strains used in this study. (DOCX)

S2 Table Plasmid used in this study. (DOCX)

S3 Table Primer used in this study. (DOCX)

S1 Fig Validation of *vipR* knockouts and determination of bacterial growth in HD-1 and HD-1Δ*vipR.* The *vipR* knockout mutant was constructed using the CRISPR-Cas9 system as described in Materials and methods. (A) PCR verification of *vipR* knockout using primers del*vipR*-upup-F/R. “del” represents the mutant gene, and “wt” represents the wild-type gene. (B) Sanger sequencing chromatogram confirming the deletion of *vipR*. (C) Growth curves of the strains constructed in this study. Primer sequences are listed in S2 Table.
(DOCX)

S2 Fig Representative microscopic images of HD-1 and HD-1Δ*vipR.* Magnified microscopic images of HD-1 and HD-1Δ*vipR* at different fermentation time points (9 h, 24 h, 36 h, and 42 h). Cells were observed under light microscopy. The scale bar represents 5 µm and applies to all panels.
(DOCX)

S3 Fig Validation of *vip3Aa* knockouts. The *vip3Aa* knockout mutant was constructed using the CRISPR-Cas9 system as described in Materials and methods. (A) PCR verification of *vip3Aa* knockout using primers del*vip3Aa*-upup-F/R. “del” represents the mutant gene, and “wt” represents the wild-type gene. (B) Sanger sequencing chromatogram confirming the deletion of *vip3Aa*. Primer sequences are listed in S2 Table.
(DOCX)

S4 Fig Identification of transcription units of insecticidal protein genes on BtPAI-1 by reverse transcription PCR (RT-PCR). (A) Schematic diagram of primer binding sites for detecting transcription units of insecticidal protein genes (*cry2Aa, cry2Ab*, *cry1Aa*, *amidase* and *cry1Ia*) located on BtPAI-1. (B-C) Agarose gel electrophoresis of RT-PCR products. (B) cDNA reverse-transcribed from total RNA of HD-1 was used as the template. (C) Genomic DNA (gDNA) was included as positive controls.
(DOCX)

S5 Fig Promoter squences of *cry2Ab*, *amidase*, *cry1Ia*, and *cry2Aa* in BtPAI-1. The -10 and -35 promoter boxes, putative VipR-binding motifs, and the start codon (ATG) are indicated. (A) Promoter squence of *cry2Ab*. (B) Promoter squence of *amidase*. (C) Promoter squence of *cry1Ia*. (D) Promoter squence of *cry2Aa*.
(DOCX)

S6 Fig Detection of *vipR* transcriptional levels in *vipR* overexpression strains. The qRT-PCR analysis of *vipR* in HD-1, CT-43, and their respective *vipR* overexpression strains (HD-1 (P_*kan*_-*vipR*), CT-43 (P_*kan*_-*vipR*),) was performed at 24 h. The *gapdh* and *16S rRNA* genes were used as internal reference genes. Data represent the means ± SD (n = 3 biological replicates). Statistical significance was determined using appropriate tests (see Materials and methods). Significance thresholds: ****P < 0.0001.
(DOCX)

S7 Fig Genomic organization of BtPAI-1 and representative BtPAI-1 variants. The figure shows the genomic arrangement of *vipR* and insecticidal protein genes within the canonical BtPAI-1 and two representative BtPAI-1 variants. The canonical BtPAI-1 is represented by strain HD-1, whereas the BtPAI-1 variants are represented by strains BGSC 4J5 and HD73, both of which lack *vipR*. Heterologous introduction of *vipR* into these strains promoted vegetative-phase transcription of *cry* genes. Arrow orientation indicates gene transcription direction, with right-pointing arrows representing genes located on the positive strand and left-pointing arrows representing genes located on the negative strand. Different colors denote different gene categories, and gene labels indicate the corresponding insecticidal proteins encoded by each locus.
(DOCX)

S8 Fig Comparative sequence analysis of BtPAI-1 and representative BtPAI-1 variants. Nucleotide sequence comparisons were performed using Easyfig v2.2.5 between the canonical BtPAI-1 from strain HD-1 and representative BtPAI-1 variants from strains BGSC 4J5 and HD73. Regions of sequence similarity are indicated by shaded areas connecting homologous loci. The analysis shows that highly homologous insecticidal protein genes are organized in distinct gene arrangements in different genomic backgrounds. Arrows indicate the positions and orientations of predicted coding sequences.
(DOCX)

S9 Fig Detection of *vipR* transcriptional levels in HD-1 at sporulation phase. The qRT-PCR analysis of *vipR* in HD-1 was performed at 24 h, 36 h and 48 h. The *gapdh* and *16S rRNA* genes were used as internal reference genes. Data represent the means ± SD (n = 3 biological replicates). Statistical significance was determined using appropriate tests (see Materials and methods). Significance thresholds: P > 0.05 (ns), ****P < 0.0001.
(DOCX)

S1 Raw Gel The raw images of the blots and gels presented in this study. This file contains the original, unprocessed scans for all key experiments. Each image is labeled according to the corresponding main or supplementary figure it supports.
(DOCX)

## References

[1] Diard M, Hardt W-D. Evolution of bacterial virulence. FEMS Microbiol Rev. 2017;41(5):679–97. doi: 10.1093/femsre/fux023 28531298

[2] Arnold DL, Jackson RW. Bacterial genomes: evolution of pathogenicity. Curr Opin Plant Biol. 2011;14(4):385–91. doi: 10.1016/j.pbi.2011.03.001 21444240

[3] Koonin EV, Makarova KS, Aravind L. Horizontal gene transfer in prokaryotes: quantification and classification. Annu Rev Microbiol. 2001;55:709–42. doi: 10.1146/annurev.micro.55.1.709 11544372 PMC4781227

[4] Soucy SM, Huang J, Gogarten JP. Horizontal gene transfer: building the web of life. Nat Rev Genet. 2015;16(8):472–82. doi: 10.1038/nrg3962 26184597

[5] Bryant JM, Brown KP, Burbaud S, Everall I, Belardinelli JM, Rodriguez-Rincon D, et al. Stepwise pathogenic evolution of *Mycobacterium abscessus*. Science. 2021;372(6541):eabb8699. doi: 10.1126/science.abb8699 33926925 PMC7611193

[6] Hacker J, Blum-Oehler G, Mühldorfer I, Tschäpe H. Pathogenicity islands of virulent bacteria: structure, function and impact on microbial evolution. Mol Microbiol. 1997;23(6):1089–97. doi: 10.1046/j.1365-2958.1997.3101672.x 9106201

[7] Hardas A, Suárez-Bonnet A, Beck S, Becker WE, Ramírez GA, Priestnall SL. Canine gastric carcinomas: a histopathological and immunohistochemical study and similarities with the human counterpart. Animals (Basel). 2021;11(5):1409. doi: 10.3390/ani11051409 34069167 PMC8156491

[8] Deng W, Puente JL, Gruenheid S, Li Y, Vallance BA, Vázquez A, et al. Dissecting virulence: systematic and functional analyses of a pathogenicity island. Proc Natl Acad Sci U S A. 2004;101(10):3597–602. doi: 10.1073/pnas.0400326101 14988506 PMC373508

[9] Gal-Mor O, Finlay BB. Pathogenicity islands: a molecular toolbox for bacterial virulence. Cell Microbiol. 2006;8(11):1707–19. doi: 10.1111/j.1462-5822.2006.00794.x 16939533

[10] Hacker J, Kaper JB. Pathogenicity islands and the evolution of microbes. Annu Rev Microbiol. 2000;54:641–79. doi: 10.1146/annurev.micro.54.1.641 11018140

[11] Cervera-Alamar M, Guzmán-Markevitch K, Žiemytė M, Ortí L, Bernabé-Quispe P, Pineda-Lucena A, et al. Mobilisation mechanism of pathogenicity islands by endogenous phages in *Staphylococcus aureus* clinical strains. Sci Rep. 2018;8(1):16742. doi: 10.1038/s41598-018-34918-2 30425253 PMC6233219

[12] Haag AF, Podkowik M, Ibarra-Chávez R, Gallego Del Sol F, Ram G, Chen J, et al. A regulatory cascade controls *Staphylococcus aureus* pathogenicity island activation. Nat Microbiol. 2021;6(10):1300–8. doi: 10.1038/s41564-021-00956-2 34518655 PMC7611864

[13] Tseng CW, Zhang S, Stewart GC. Accessory gene regulator control of staphyloccoccal enterotoxin d gene expression. J Bacteriol. 2004;186(6):1793–801. doi: 10.1128/JB.186.6.1793-1801.2004 14996810 PMC355899

[14] Schmidt KA, Donegan NP, Kwan WA Jr, Cheung A. Influences of sigmaB and agr on expression of staphylococcal enterotoxin B (seb) in *Staphylococcus aureus*. Can J Microbiol. 2004;50(5):351–60. doi: 10.1139/w04-017 15213743

[15] Maiques E, Quiles-Puchalt N, Donderis J, Ciges-Tomas JR, Alite C, Bowring JZ, et al. Another look at the mechanism involving trimeric dUTPases in *Staphylococcus aureus* pathogenicity island induction involves novel players in the party. Nucleic Acids Res. 2016;44(11):5457–69. doi: 10.1093/nar/gkw317 27112567 PMC4914113

[16] Bravo A, Likitvivatanavong S, Gill SS, Soberón M. *Bacillus thuringiensis*: a story of a successful bioinsecticide. Insect Biochem Mol Biol. 2011;41(7):423–31. doi: 10.1016/j.ibmb.2011.02.006 21376122 PMC3689885

[17] Shi J, Sun M. Bacillus thuringiensis: a gift for nematode management. Trends Parasitol. 2025;41(3):235–46. doi: 10.1016/j.pt.2025.01.004 39939273

[18] He J, Wang J, Yin W, Shao X, Zheng H, Li M, et al. Complete genome sequence of *Bacillus thuringiensis* subsp. *chinensis* strain CT-43. J Bacteriol. 2011;193(13):3407–8. 21551307 10.1128/JB.05085-11 PMC3133296

[19] Zheng J, Gao Q, Liu L, Liu H, Wang Y, Peng D, et al. Comparative genomics of *Bacillus thuringiensis* reveals a path to specialized exploitation of multiple invertebrate hosts. mBio. 2017;8(4):e00822-17. doi: 10.1128/mBio.00822-17 28790205 PMC5550751

[20] Adams LF, Brown KL, Whiteley HR. Molecular cloning and characterization of two genes encoding sigma factors that direct transcription from a *Bacillus thuringiensis* crystal protein gene promoter. J Bacteriol. 1991;173(12):3846–54. doi: 10.1128/jb.173.12.3846-3854.1991 1904859 PMC208016

[21] Zhang J, Schairer HU, Schnetter W, Lereclus D, Agaisse H. Bacillus popilliae cry18Aa operon is transcribed by sigmaE and sigmaK forms of RNA polymerase from a single initiation site. Nucleic Acids Res. 1998;26(5):1288–93. doi: 10.1093/nar/26.5.1288 9469839 PMC147395

[22] Poncet S, Dervyn E, Klier A, Rapoport G. Spo0A represses transcription of the cry toxin genes in Bacillus thuringiensis. Microbiology (Reading). 1997;143 (Pt 8):2743–51. doi: 10.1099/00221287-143-8-2743 9274027

[23] Wang Y, Deng C, Peng Q, Chen Z, Huang D, Zhang J, et al. Effect of quorum sensing response regulator nprR deletion on expression of cry protein in Bacillus thuringiensis. Wei Sheng Wu Xue Bao. 2010;50(11):1550–5. 21268903

[24] Dubois T, Perchat S, Verplaetse E, Gominet M, Lemy C, Aumont-Nicaise M, et al. Activity of the Bacillus thuringiensis NprR-NprX cell-cell communication system is co-ordinated to the physiological stage through a complex transcriptional regulation. Mol Microbiol. 2013;88(1):48–63. doi: 10.1111/mmi.12168 23388036

[25] Hou X, Mao C, Zhang W, Jiang L, Li M, Guo J, et al. Structural and functional roles of domain III in Vip3Aa and Vip3Ca: implications for membrane perforation and insecticidal efficacy. Pest Manag Sci. 2025;81(9):5686–95. doi: 10.1002/ps.8922 40411233

[26] Park Y, Hua G, Ambati S, Taylor M, Adang MJ. Binding and synergizing motif within coleopteran cadherin enhances Cry3Bb toxicity on the *Colorado Potato Beetle* and the *Lesser Mealworm*. Toxins (Basel). 2019;11(7):386. doi: 10.3390/toxins11070386 31269670 PMC6669875

[27] Estruch JJ, Warren GW, Mullins MA, Nye GJ, Craig JA, Koziel MG. Vip3A, a novel Bacillus thuringiensis vegetative insecticidal protein with a wide spectrum of activities against lepidopteran insects. Proc Natl Acad Sci U S A. 1996;93(11):5389–94. doi: 10.1073/pnas.93.11.5389 8643585 PMC39256

[28] Syed T, Askari M, Meng Z, Li Y, Abid MA, Wei Y, et al. Current insights on vegetative insecticidal proteins (Vip) as next generation pest killers. Toxins (Basel). 2020;12(8):522. doi: 10.3390/toxins12080522 32823872 PMC7472478

[29] Agaisse H, Lereclus D. Structural and functional analysis of the promoter region involved in full expression of the cryIIIA toxin gene of Bacillus thuringiensis. Mol Microbiol. 1994;13(1):97–107. doi: 10.1111/j.1365-2958.1994.tb00405.x 7984098

[30] Zhang X, Gao T, Peng Q, Song L, Zhang J, Chai Y, et al. A strong promoter of a non-cry gene directs expression of the cry1Ac gene in *Bacillus thuringiensis*. Appl Microbiol Biotechnol. 2018;102(8):3687–99. doi: 10.1007/s00253-018-8836-5 29520600

[31] Singh AK, Paritosh K, Kant U, Burma PK, Pental D. High expression of Cry1Ac protein in Cotton (*Gossypium hirsutum*) by combining independent transgenic events that target the protein to cytoplasm and plastids. PLoS One. 2016;11(7):e0158603. doi: 10.1371/journal.pone.0158603 27391960 PMC4938423

[32] Deng C, Slamti L, Raymond B, Liu G, Lemy C, Gominet M, et al. Division of labour and terminal differentiation in a novel *Bacillus thuringiensis* strain. ISME J. 2015;9(2):286–96. doi: 10.1038/ismej.2014.122 25083932 PMC4303623

[33] Zhang R, Slamti L, Tong L, Verplaetse E, Ma L, Lemy C, et al. The stationary phase regulator CpcR activates cry gene expression in non-sporulating cells of *Bacillus thuringiensis*. Mol Microbiol. 2020;113(4):740–54. doi: 10.1111/mmi.14439 31793098

[34] Chen H, Verplaetse E, Slamti L, Lereclus D. Expression of the *Bacillus thuringiensis* vip3A insecticidal toxin gene is activated at the onset of stationary phase by VipR, an autoregulated transcription factor. Microbiol Spectr. 2022;10(4):e0120522. doi: 10.1128/spectrum.01205-22 35727045 PMC9430311

[35] Donovan WP, Donovan JC, Engleman JT. Gene knockout demonstrates that vip3A contributes to the pathogenesis of *Bacillus thuringiensis* toward *Agrotis ipsilon* and *Spodoptera exigua*. J Invertebr Pathol. 2001;78(1):45–51. doi: 10.1006/jipa.2001.5037 11500093

[36] Noviello G. Transcriptional regulation as a dose-dependent process: insights from transcription factor tuning. Open Biol. 2025;15(8):240328. doi: 10.1098/rsob.240328 40763802 PMC12324873

[37] Cardoso P, Fazion F, Perchat S, Buisson C, Vilas-Bôas G, Lereclus D. Rap-Phr systems from plasmids pAW63 and pHT8-1 Act together to regulate sporulation in the *Bacillus thuringiensis* Serovar *kurstaki* HD73 strain. Appl Environ Microbiol. 2020;86(18):e01238-20. doi: 10.1128/AEM.01238-20 32680861 PMC7480360

[38] Gómez I, Garcia-Gómez BI, do Nascimento NA, Infante O, Cantón PE, Pacheco S, et al. Regulatory diversity in *Bacillus thuringiensis* cry genes reveals flexible evolutionary strategies for in vivo toxin expression. PLoS Pathog. 2026;22(3):e1014017. doi: 10.1371/journal.ppat.1014017 41779819 PMC12970973

[39] Liu H, Zheng J, Bo D, Yu Y, Ye W, Peng D, et al. BtToxin_Digger: a comprehensive and high-throughput pipeline for mining toxin protein genes from *Bacillus thuringiensis*. Bioinformatics. 2021;38(1):250–1. doi: 10.1093/bioinformatics/btab506 34244720

[40] Grant CE, Bailey TL, Noble WS. FIMO: scanning for occurrences of a given motif. Bioinformatics. 2011;27(7):1017–8. doi: 10.1093/bioinformatics/btr064 21330290 PMC3065696

[41] Tambassi M, Berni M, Bracchi C, Menozzi I, Dodi A, Mazzera L, et al. *Salmonella* pathogenicity Island 1 undergoes decay in serovars adapted to swine and poultry. Microbiol Spectr. 2025;13(1):e0264324. doi: 10.1128/spectrum.02643-24 39660884 PMC11705904

[42] Snoeck S, Guidi C, De Mey M. “Metabolic burden” explained: stress symptoms and its related responses induced by (over)expression of (heterologous) proteins in *Escherichia coli*. Microb Cell Fact. 2024;23(1):96. doi: 10.1186/s12934-024-02370-9 38555441 PMC10981312

[43] Ma R, Liu Y, Gan J, Qiao H, Ma J, Zhang Y, et al. Xenogeneic nucleoid-associated EnrR thwarts H-NS silencing of bacterial virulence with unique DNA binding. Nucleic Acids Res. 2022;50(7):3777–98. doi: 10.1093/nar/gkac180 35325196 PMC9023278

[44] Verplaetse E, Slamti L, Lereclus D. Paradigm shift for *cry* gene expression in *Bacillus thuringiensis*. bioRxiv. 2025:2025.06.13.659657. 10.1128/mbio.01592-26 PMC13556251 42554477

[45] Jeong H, Barbe V, Lee CH, Vallenet D, Yu DS, Choi S-H, et al. Genome sequences of *Escherichia coli* B strains REL606 and BL21(DE3). J Mol Biol. 2009;394(4):644–52. doi: 10.1016/j.jmb.2009.09.052 19786035

[46] Sezonov G, Joseleau-Petit D, D’Ari R. Escherichia coli physiology in Luria-Bertani broth. J Bacteriol. 2007;189(23):8746–9. doi: 10.1128/JB.01368-07 17905994 PMC2168924

[47] AM D. Molecular cloning: a laboratory manual. 1983;49(2):411 p.

[48] Peng D, Luo Y, Guo S, Zeng H, Ju S, Yu Z, et al. Elaboration of an electroporation protocol for large plasmids and wild-type strains of *Bacillus thuringiensis*. J Appl Microbiol. 2009;106(6):1849–58. doi: 10.1111/j.1365-2672.2009.04151.x 19291242

[49] Capella-Gutiérrez S, Silla-Martínez JM, Gabaldón T. trimAl: a tool for automated alignment trimming in large-scale phylogenetic analyses. Bioinformatics. 2009;25(15):1972–3. doi: 10.1093/bioinformatics/btp348 19505945 PMC2712344

[50] Edgar RC. MUSCLE: multiple sequence alignment with high accuracy and high throughput. Nucleic Acids Res. 2004;32(5):1792–7. doi: 10.1093/nar/gkh340 15034147 PMC390337

[51] Li L, Stoeckert CJ Jr, Roos DS. OrthoMCL: identification of ortholog groups for eukaryotic genomes. Genome Res. 2003;13(9):2178–89. doi: 10.1101/gr.1224503 12952885 PMC403725

[52] Letunic I, Bork P. Interactive Tree Of Life (iTOL): an online tool for phylogenetic tree display and annotation. Bioinformatics. 2007;23(1):127–8. doi: 10.1093/bioinformatics/btl529 17050570

[53] Jiang Y, Chen B, Duan C, Sun B, Yang J, Yang S. Multigene editing in the *Escherichia coli* genome via the CRISPR-Cas9 system. Appl Environ Microbiol. 2015;81(7):2506–14. doi: 10.1128/AEM.04023-14 25636838 PMC4357945

[54] Blin K, Pedersen LE, Weber T, Lee SY. CRISPy-web: an online resource to design sgRNAs for CRISPR applications. Synth Syst Biotechnol. 2016;1(2):118–21. doi: 10.1016/j.synbio.2016.01.003 29062934 PMC5640694

[55] Altenbuchner J. Editing of the *Bacillus subtilis* genome by the CRISPR-Cas9 system. Appl Environ Microbiol. 2016;82(17):5421–7. doi: 10.1128/AEM.01453-16 27342565 PMC4988203

[56] Day M, Ibrahim M, Dyer D, Bulla L Jr. Genome Sequence of *Bacillus thuringiensis* subsp. kurstaki Strain HD-1. Genome Announc. 2014;2(4):e00613-14. doi: 10.1128/genomeA.00613-14 25035322 PMC4102859

[57] Sun M, Yu Z. Characterization of insecticidal crystal proteins of *Bacillus thuringiensis* subsp. chinensis CT-43. Wei Sheng Wu Xue Bao. 1996;36(4):303–6. 9639832

[58] Pasini A, Parra JR, Lopes JM. Artificial diet for rearing Doru luteipes (Scudder) (Dermaptera: Forficulidae), a predator of the fall armyworm, *Spodoptera frugiperda* (J.E. Smith) (Lepidoptera: Noctuidae). Neotrop Entomol. 2007;36(2):308–11. 17607467 10.1590/s1519-566x2007000200020

[59] Gutierrez-Moreno R, Mota-Sanchez D, Blanco CA, Chandrasena D, Difonzo C, Conner J, et al. Susceptibility of fall armyworms (*Spodoptera frugiperda* J.E.) from mexico and puerto rico to Bt proteins. Insects. 2020;11(12). 33255898 10.3390/insects11120831 PMC7760814

[60] Wahiduzzaman, Dar MA, Haque MA, Idrees D, Hassan MI, Islam A, et al. Characterization of folding intermediates during urea-induced denaturation of human carbonic anhydrase II. Int J Biol Macromol. 2017;95:881–7. doi: 10.1016/j.ijbiomac.2016.10.073 27789330

[61] Reese MG. Application of a time-delay neural network to promoter annotation in the *Drosophila melanogaster* genome. Comput Chem. 2001;26(1):51–6. doi: 10.1016/s0097-8485(01)00099-7 11765852

[62] Arantes O, Lereclus D. Construction of cloning vectors for *Bacillus thuringiensis*. Gene. 1991;108(1):115–9. doi: 10.1016/0378-1119(91)90495-w 1662180

[63] Grandvalet C, Gominet M, Lereclus D. Identification of genes involved in the activation of the *Bacillus thuringiensis* inhA metalloprotease gene at the onset of sporulation. Microbiology (Reading). 2001;147(Pt 7):1805–13. doi: 10.1099/00221287-147-7-1805 11429458

