# Supplementary material for: Bacillus thuringiensis pathogenicity islands encode regulatory circuits controlling insecticidal Cry toxin expression during vegetative growth
Source: PLoS Pathog. 2026 Jul 29;22(7):e1014476. doi: 10.1371/journal.ppat.1014476 (PMC13440803; doi:10.1371/journal.ppat.1014476)
Supplement: Supplementary file 1 [file ppat.1014476.s001.docx]

**S1 Table. Strains used in this study.**

| Strain | Description | Source or reference |
| --- | --- | --- |
| HD-1 | *Bacillus thuringiensis* serovar *kurstaki* HD-1. This strain was designated as the wild-type strain and was used to construct the other strains described below. | 57 |
| HD73 | *Bacillus thuringiensis* serovar *kurstaki* HD73. This strain was designated as the wild-type strain and was used to construct the other strains described below. | 37 |
| CT-43 | *Bacillus thuringiensis* serovar *chinensis* CT-43. This strain was designated as the wild-type strain and was used to construct the other strains described below. | 58 |
| BGSC 4J5 | *Bacillus thuringiensis* *serovar* *aizawai* BGSC 4J5. This strain was designated as the wild-type strain and was used to construct all the other strains described below. | 19 |
| BGSC 4AE1 | *Bacillus thuringiensis* serovar *amagiensis* BGSC 4AE1. This strain was designated as the wild-type strain and was used to construct all the other strains described below. | 19 |
| *vipR*-pJOE8999/HD-1 | The *vipR*-pJOE8999 plasmid was transformed into HD-1 strain for gene knockout. | This study |
| *vip3Aa*-pJOE8999/HD-1 | The *vip3Aa*-pJOE8999 plasmid was transformed into HD-1 strain for gene knockout. | This study |
| HD-1Δ*vipR* | HD-1 with *vipR* deleted, constructed using CRISPR-Cas9 | This study |
| HD-1Δ*vip3Aa* | HD-1 with *vip3Aa* deleted, constructed using CRISPR-Cas9 | This study |
| HD-1(P*_cry2Ab_*-lacZ) | HD-1 carrying the transcription fusion plasmid pHT304-*P_cry2Ab_*-lacZ. | This study |
| HD-1(P*_cry2Aa_*-lacZ) | HD-1 carrying the transcription fusion plasmid pHT304-*P_cry2Aa_*-lacZ. | This study |
| HD-1(P*_amidase_*-lacZ) | HD-1 carrying the transcription fusion plasmid pHT304-*P_amidase_*-lacZ. | This study |
| HD-1(P*_cry1Ia_*-lacZ) | HD-1 carrying the transcription fusion plasmid pHT304-*P_cry1Ia_*-lacZ. | This study |
| HD-1Δ*vipR*(P*_cry2Ab_*-lacZ) | HD-1Δ*vipR* carrying the transcription fusion plasmid pHT304-*P_cry2Ab_*-lacZ. | This study |
| HD-1Δ*vipR*(P*_cry2Aa_*-lacZ) | HD-1Δ*vipR* carrying the transcription fusion plasmid pHT304-*P_cry2Aa_*-lacZ. | This study |
| HD-1Δ*vipR*(P*_amidase_*-lacZ) | HD-1Δ*vipR* carrying the transcription fusion plasmid pHT304-*P_amidase_*-lacZ. | This study |
| HD-1Δ*vipR*(P*_cry1Ia_*-lacZ) | HD-1Δ*vipR* carrying the transcription fusion plasmid pHT304-*P_cry1Ia_*-lacZ. | This study |
| HD73(*vipR*) | HD73 carring the plasmid pHT304-*vipR* | This study |
| BGSC 4J5(*vipR*) | BGSC 4J5 carring the plasmid pHT304-*vipR* | This study |
| BGSC 4AE1(*vipR*) | BGSC 4AE1 carring the plasmid pHT304-*vipR* | This study |
| HD-1(P*_kan_*-*vipR*) | HD-1 carrying the *vipR*-overexpressing plasmid pHT315-P*_kan_*-*vipR.* | This study |
| CT-43(P*_kan_*-*vipR*) | CT-43 carrying the *vipR*-overexpressing plasmid pHT315-P*_kan_*-*vipR.* | This study |
