# Supplementary material for: Bacillus thuringiensis pathogenicity islands encode regulatory circuits controlling insecticidal Cry toxin expression during vegetative growth
Source: PLoS Pathog. 2026 Jul 29;22(7):e1014476. doi: 10.1371/journal.ppat.1014476 (PMC13440803; doi:10.1371/journal.ppat.1014476)
Supplement: Supplementary file 2 [file ppat.1014476.s002.docx]

**S2 Table. Plasmid used in this study.**

| Short name | Description | Source or reference |
| --- | --- | --- |
| pJOE8999 | Contains CRISPR-Cas9 system plasmid for breaking genome; Kanamycin(100μg/ml) | 56 |
| pHT304-18Z | *E. coli*/*B. thuringiensis* shuttle vector conferring resistance to ampicillin in *E. coli* and to erythromycin in *B. thuringiensis.* Designed to allow transcriptional fusion with *lacZ* (59) | 63 |
| *vipR*-pJOE8999 | This plasmid contained the sgRNA targeting *vipR* along with the upstream and downstream homologous arms for gene knockout. | This study |
| *vip3Aa*-pJOE8999 | This plasmid contained the sgRNA targeting *vip3Aa* along with the upstream and downstream homologous arms for gene knockout | This study |
| pHT304-P*_vip3Aa_*-*lacZ* | A 413-bp *BamH*I-*Hind*III fragment containing the promoter of *vip3Aa* was PCR amplified using genomic DNA of the HD-1 strain as the template. The DNA fragment was ligated between the *BamH*I*-Hind*III sites of pHT304-18Z | This study |
| pHT304-P*_cry2Ab_*-*lacZ* | A 455-bp *BamH*I-*Hind*III fragment containing the promoter of *cry2Ab* was PCR amplified using genomic DNA of the HD-1 as the template. The DNA fragment was ligated between the *BamH*I-*Hind*III sites of pHT304-18Z | This study |
| pHT304-P*_amidase_*-*lacZ* | A 347-bp *BamH*I-*Hind*III fragment containing the promoter of *amidase* was PCR amplified using genomic DNA of the HD-1 as the template. The DNA fragment was ligated between the *BamH*I-*Hind*III sites of pHT304-18Z | This study |
| pHT304-P*_cry1Ia_*-*lacZ* | A 457-bp *BamH*I-*Hind*III fragment containing the promoter of *cry1Ia* was PCR amplified using genomic DNA of the HD-1 as the template. The DNA fragment was ligated between the *BamH*I-*Hind*III sites of pHT304-18Z | This study |
| pHT304-P*_vipR_*-*vipR* | A 2,644-bp DNA fragment containing the promoter of vipR and the *vipR* coding sequence was PCR amplified using genomic DNA of the HD-1 strain as the template. This DNA fragment was ligated in the *BamH*I-*Hind*III sites of pHT304. | This study |
| pHT315 | *E. coli*//*B. thuringiensis* shuttle vector conferring resistance to ampicillin in *E. coli* and to erythromycin in *B. thuringiensis*. This plasmid carries the constitutive promoter P_kan_, which significantly enhances the transcriptional level of genes. | 43 |
| pHT315-P*_kan_*-*vipR* | A 1,416-bp DNA fragment containing the *vipR* coding sequence was PCR amplified using genomic DNA of the HD-1 strain as the template. This DNA fragment was ligated in the *Xba*I-*Hind*III sites of pHT315. | This study |
