# Supplementary material for: Bacillus thuringiensis pathogenicity islands encode regulatory circuits controlling insecticidal Cry toxin expression during vegetative growth
Source: PLoS Pathog. 2026 Jul 29;22(7):e1014476. doi: 10.1371/journal.ppat.1014476 (PMC13440803; doi:10.1371/journal.ppat.1014476)
Supplement: Supplementary file 3 [file ppat.1014476.s003.docx]

**S3 Table. Primer used in this study.**

| Primer | Sequence(5’-3’) | Functions |
| --- | --- | --- |
| del*vipR*-sgRNA-F | TACGTATTTCATTGCTATCAATCA | To provide guide RNA for the construction of HD-1Δ*vipR*. |
| del*vipR*-sgRNA-F | AAACTGATTGATAGCAATGAAATA |  |
| del*vipR*-sfiI-up-F | AAGGCCAACGAGGCCGGAAGGAGTTATAAGGATCGA | To provide the upstream homologous repair fragment for constructing the HD-1Δ*vipR*. |
| del*vipR*-sfiI-up-R | AAGGCCATGTTGGCCCATTTCTCCTCCCTTAATACTG |  |
| del*vipR*-sfiI-down-R | AAGGCCAACATGGCCCCCAATACCTATAATTCGGATC | To provide the upstream homologous repair fragment for constructing the HD-1Δ*vipR*. |
| del*vipR*-sfiI-down-R | AAGGCCTTATTGGCCGCATTCGAAAGCATTAATCG |  |
| del*vipR*-upup-F | TGCCGTATTTCTTAGTATGC | For verifying the knockout result of *vipR*. |
| del*vipR*-upup-R | CACGTCAATACACTATTGGTAG |  |
| del*vipR*-dodo-F | GTTATGTAAACATGGGTGCTC | For verifying the knockout result of *vipR*. |
| del*vipR*-dodo-R | ACGCTCCTTGAGGAAAGA |  |
| del*vip3Aa*-sgRNA-F | TACGATTCTTTAAAATTTCGTCTA | To provide guide RNA for the construction of HD-1Δ*vip3Aa* |
| del*vip3Aa*-sgRNA-F | AAACTAGACGAAATTTTAAAGAAT |  |
| del*vip3Aa*-sfiI-up-F | AAGGCCAACGAGGCC gatcgattatatttatagg | To provide the upstream homologous repair fragment for constructing the HD-1Δ*vip3Aa*. |
| del*vip3Aa*-sfiI-up-R | AAGGCCATGTTGGCC gttcatcttccttttcagc |  |
| del*vip3Aa*-sfiI-down-R | AAGGCCAACATGGCC gatcgggatctaatattaac | To provide the upstream homologous repair fragment for constructing the HD-1Δ*vip3Aa*. |
| del*vip3Aa*-sfiI-down-R | AAGGCCTTATTGGCC cgtctgatattagtaaggac |  |
| del*vip3Aa*-upup-F | CGTAACGATTAGCCGAGAA | For verifying the knockout result of *vip3Aa*. |
| del*vip3Aa*-upup-R | ACCTACTCATTGTGCTGTG |  |
| del*vip3Aa*-dodo-F | AACAGGTGATAGCATCCAAT | For verifying the knockout result of *vip3Aa*. |
| del*vip3Aa*-dodo-R | GAACAAGCCAATTCATCTCC |  |
| qRT-*vip3A*-F | TATCGCACAGGGAAACTTA | To analysis the transcription of *vip3Aa* mRNA in different strains by qRT-PCR. |
| qRT-*vip3A*-R | ACATCACTCAACATAGAGGT |  |
| qRT-*cry2Ab*-F | TTCAGCAGCAACATTACG | To analysis the transcription of *cry2Ab* mRNA in different strains by qRT-PCR. |
| qRT-*cry2Ab*-R | ACAACGACCAGATAGATACA |  |
| qRT-*cry9Aa*-F | CAGATATTATGGACGCTCAA | To analysis the transcription of *cry9Aa* mRNA in different strains by qRT-PCR. |
| qRT-*cry9Aa*-R | CAGATACGATGCTTGTTGT |  |
| Cas9-F | TTCGTTATCTTCTGGACTACC | To verify the presence of the pHT315-Cas9 plasmid. |
| Cas9-R | TCGTTGGAACTGCTTTGAT |  |
| qRT-*amidase*-F | GGTGGATCATCTGGTGTAT | To analysis the transcription of amidase mRNA in different strains by qRT-PCR. |
| qRT-*amidase*-R | CCGATAGACGAGCGATTA |  |
| qRT-*cry1Aa*-F | GTTCGGTCCTTGTTGTTC | To analysis the transcription of *cry1Aa* mRNA in different strains by qRT-PCR. |
| qRT-*cry1Aa*-R | TCCTCTTCTACGCAGTTG |  |
| qRT-*cry1Ia*-F | ATTCGCTATGCTTCTACCA | To analysis the transcription of *cry1Ia* mRNA by qRT-PCR. |
| qRT-*cry1Ia*-R | AGTCTAAGTCCTCTCCTCTAT |  |
| pJOE8999-F | CAATGGACAATGGACACCTA | For verifying the presence of plasmid pJOE8999. |
| pJOE8999-R | TGCCACTCTTATCCATCAAT |  |
| qRT-*cry2Aa*-F | TCCAGCCAACTTCTAACA | To analysis the transcription of *cry2Aa* mRNA in different strains by qRT-PCR. |
| qRT-*cry2Aa*-R | GCGACTAATCTCAATCACAA |  |
| RT-*cry2Aa*-1-F | TAAATAAATCCAACATTGG | To clarify the transcriptional unit where *cry2Aa* is located. |
| RT-*cry2Aa*-1-R | TTTGTGCCAACTAATCTTCC |  |
| RT-*cry2Aa*-2-F | GCTTTGTTCAAATCTTAAG |  |
| RT-*cry2Aa*-2-R | TAGCGGAGGAGTTTCATCTGG |  |
| RT-*cry2Aa*-3-F | CAATACATTATTCATATAAA |  |
| RT-*cry2Aa*-3-R | CTCAAAGCCGCAGGAAAAGAG |  |
| RT-*cry2Ab*-1-F | GGATTCTCTAATACACCTGT | To clarify the transcriptional unit where *cry2Ab* is located. |
| RT-*cry2Ab*-1-R | AAGCCTACATTTAGCCATC |  |
| RT-*cry2Ab*-2-F | GTGGTGTCTCATCTGGTATA |  |
| RT-*cry2Ab*-2-R | AGTAGTTCTTCCGCTATTCA |  |
| RT-*cry2Ab*-3-F | ACCAACTAATATTTCACCA |  |
| RT-*cry2Ab*-3-R | TGCAATTTTGACATAAATACTC |  |
| RT-ami-2-F | TTTAGGGAAATAAAATCTT | To clarify the transcriptional unit where amidase is located. |
| RT-*ami*-2-R | ACTGTCATATAGCTACATCTCC |  |
| RT-*ami*-3-F | GGTGGATTTGAGGGTTTCAA |  |
| RT-*ami*-3-R | TTCCACGAAAACTACCATC |  |
| RT-*cry1Aa*-1-F | CCAAATAATCAGGAACTGTTT | To clarify the transcriptional unit where *cry1Aa* is located. |
| RT-*cry1Aa*-1-R | AACGACAGAAGTTCCAGAGCC |  |
| RT-*cry1Aa*-2-F | TCCAAATAACACGGTAACGTG |  |
| RT-*cry1Aa*-2-R | ATCTCTGCTTGATTAAGTTG |  |
| RT-*cry1Ia*-1-F | ATTTATCGTGGACAGCGTG | To clarify the transcriptional unit where *cry1Ia* is located. |
| RT-*cry1Ia*-1-R | AGAGATTTTATCTACTTTCG |  |
| RT-*cry1Ia*-2-F | AGGATTCAGAAAATGAATT |  |
| RT-*cry1Ia*-2-R | ATAGTAAATAGAGAGCATT |  |
| VipR-15D-F | CGCCATATGGATAAGTTCATCACCGATCT | For constructing the *vipR* expression vector. |
| VipR-15D-R | CCGCTCGAGTTAATTAAACGGACTCAGAA |  |
| EMSA-*vip3Aa*-F | taacctttggtacaattcg | A 413-bp fragment containing the *vip3Aa* promoter region was used as the nucleic acid probe for EMSA. The forward primer was labeled with *FAM* to facilitate subsequent chemiluminescence detection. |
| EMSA-*vip3Aa*-R | ctttctctggggctatgag |  |
| EMSA-*cry2Ab*-F | ACTGCGGAGATAGCCTAA | A 455-bp fragment containing the *cry2Ab* promoter region was used as the nucleic acid probe for EMSA. The forward primer was labeled with *FAM* to facilitate subsequent chemiluminescence detection. |
| EMSA-*cry2Ab*-R | AGTATAAAATTTGAAAACTG |  |
| EMSA-*ami*-F | GCATGAATTGAGGGATTGA | A 347-bp fragment containing the *amidase* promoter region was used as the nucleic acid probe for EMSA. The forward primer was labeled with *FAM* to facilitate subsequent chemiluminescence detection. |
| EMSA-*ami*-R | GCTACATCTCCTATCAGATT |  |
| EMSA-*cry1Ia*-F | TCCAAGAGCAGCATTACAA | A 457-bp fragment containing the *cry1Ia* promoter region was used as the nucleic acid probe for EMSA. The forward primer was labeled with *FAM* to facilitate subsequent chemiluminescence detection. |
| EMSA-*cry1Ia*-R | CCCTCCACTTATACTATTAT |  |
| Lacz-*vip3Aa*-F | TGCACTGCAGtaacctttggtacaattcg | A 413-bp *BamH*I-*Hind*III fragment containing the promoter of *vip3Aa* for β-Galactosidase activity assay. |
| Lacz-*vip3Aa*-R | CGCGGATCCctttctctggggctatgag |  |
| Lacz-*cry2Ab*-F | TGCACTGCAG GCATGAATTGAGGGATTGA | A 455-bp *BamH*I-*Hind*III fragment containing the promoter of *cry2Ab* for β-Galactosidase activity assay. |
| Lacz-*cry2Ab*-R | CGCGGATCC ATAGCTACATCTCCTATCAG |  |
| Lacz-*ami*-F | TGCACTGCAG TCCAAGAGCAGCATTACAA | A 347-bp *BamH*I-*Hind*III fragment containing the promoter of *amidase* for β-Galactosidase activity assay. |
| Lacz-*ami*-R | CGCGGATCC ATTAATCCCTCCACTTATAC |  |
| Lacz-*cry1Ia*-F | TGCACTGCAG ACTGCGGAGATAGCCTAA | A 457-bp *BamH*I-*Hind*III fragment containing the promoter of *cry1Ia* for β-Galactosidase activity assay. |
| Lacz-*cry1Ia*-R | CGCGGATCC AGTATAAAATTTGAAAACTG |  |
| P*_vipR_*-*vipR*-F | TGCTCTAGAGTTATAGAGGAATTAGATAA | A 2,644-bp DNA fragment containing the promoter of *vipR* and the *vipR* coding sequence was used to transfer *vipR* into strains lacking *vipR*. |
| P*_vipR_*-*vipR*-R | CCCAAGCTTGAAATTGCATTCAAAAGCA |  |
| P*_kan_*-*vipR*-F | TTAGCTTCTTGGGGTCTAGAatggataaatttattacaga | A 1,416-bp DNA fragment containing the *vipR* coding sequence was used to construct overexpression strains. |
| P*_kan_*-*vipR*-R | ACCATGATTACGCCAAGCTTttagttaaaaggggataaaac |  |
