# Supplementary material for: Bacillus thuringiensis pathogenicity islands encode regulatory circuits controlling insecticidal Cry toxin expression during vegetative growth
Source: PLoS Pathog. 2026 Jul 29;22(7):e1014476. doi: 10.1371/journal.ppat.1014476 (PMC13440803; doi:10.1371/journal.ppat.1014476)
Supplement: Supplementary file 5 [file ppat.1014476.s005.docx]

**S2 Fig. Representative microscopic images of HD-1 and HD-1Δ*vipR.***

Magnified microscopic images of HD-1 and HD-1Δ*vipR* at different fermentation time points (9 h, 24 h, 36 h, and 42 h). Cells were observed under light microscopy. The scale bar represents 5 µm and applies to all panels.
