# Supplementary material for: Bacillus thuringiensis pathogenicity islands encode regulatory circuits controlling insecticidal Cry toxin expression during vegetative growth
Source: PLoS Pathog. 2026 Jul 29;22(7):e1014476. doi: 10.1371/journal.ppat.1014476 (PMC13440803; doi:10.1371/journal.ppat.1014476)
Supplement: Supplementary file 6 [file ppat.1014476.s006.docx]

**S3 Fig. Validation of *vip3Aa* knockouts.**

The *vip3Aa* knockout mutant was constructed using the CRISPR-Cas9 system as described in Materials and Methods. (A) PCR verification of *vip3Aa* knockout using primers del*vip3Aa*-upup-F/R. "del" represents the mutant gene, and "wt" represents the wild-type gene. (B) Sanger sequencing chromatogram confirming the deletion of *vip3Aa*. Primer sequences are listed in S2 Table.
