# Supplementary material for: Bacillus thuringiensis pathogenicity islands encode regulatory circuits controlling insecticidal Cry toxin expression during vegetative growth
Source: PLoS Pathog. 2026 Jul 29;22(7):e1014476. doi: 10.1371/journal.ppat.1014476 (PMC13440803; doi:10.1371/journal.ppat.1014476)
Supplement: Supplementary file 7 [file ppat.1014476.s007.docx]

**S4 Fig. Identification of transcription units of insecticidal protein genes on BtPAI-1 by reverse transcription PCR (RT-PCR).**

(A) Schematic diagram of primer binding sites for detecting transcription units of insecticidal protein genes (*cry2Aa, cry2Ab*, *cry1Aa*, *amidase* and *cry1Ia*) located on BtPAI-1. (B-C) Agarose gel electrophoresis of RT-PCR products. (B) cDNA reverse-transcribed from total RNA of HD-1 was used as the template. (C) Genomic DNA (gDNA) was included as positive controls.
