# Supplementary material for: Bacillus thuringiensis pathogenicity islands encode regulatory circuits controlling insecticidal Cry toxin expression during vegetative growth
Source: PLoS Pathog. 2026 Jul 29;22(7):e1014476. doi: 10.1371/journal.ppat.1014476 (PMC13440803; doi:10.1371/journal.ppat.1014476)
Supplement: Supplementary file 8 [file ppat.1014476.s008.docx]

**S5 Fig. Promoter squences of *cry2Ab*, *amidase*, *cry1Ia*, and *cry2Aa* in BtPAI-1.**

The -10 and -35 promoter boxes, putative VipR-binding motifs, and the start codon (ATG) are indicated. (A) Promoter sequence of *cry2Ab*. (B) Promoter sequence of *amidase*. (C) Promoter sequence of *cry1Ia*. (D) Promoter sequence of *cry2Aa*.
