# Supplementary material for: Bacillus thuringiensis pathogenicity islands encode regulatory circuits controlling insecticidal Cry toxin expression during vegetative growth
Source: PLoS Pathog. 2026 Jul 29;22(7):e1014476. doi: 10.1371/journal.ppat.1014476 (PMC13440803; doi:10.1371/journal.ppat.1014476)
Supplement: Supplementary file 9 [file ppat.1014476.s009.docx]

**S6 Fig.** **Detection of *vipR* transcriptional levels in *vipR* overexpression strains.**

The qRT-PCR analysis of *vipR* in HD-1, CT-43, and their respective *vipR* overexpression strains (HD-1 (P*_kan_*-*vipR*), CT-43 (P*_kan_*-*vipR*),) was performed at 24 h. The *gapdh* and *16S rRNA* genes were used as internal reference genes. Data represent the means ± SD (n = 3 biological replicates). Statistical significance was determined using appropriate tests (see Materials and Methods). Significance thresholds: ****P < 0.0001.
