# Supplementary material for: Bacillus thuringiensis pathogenicity islands encode regulatory circuits controlling insecticidal Cry toxin expression during vegetative growth
Source: PLoS Pathog. 2026 Jul 29;22(7):e1014476. doi: 10.1371/journal.ppat.1014476 (PMC13440803; doi:10.1371/journal.ppat.1014476)
Supplement: Supplementary file 10 [file ppat.1014476.s010.docx]

**S7 Fig. Genomic organization of BtPAI-1 and representative BtPAI-1 variants.**

The figure shows the genomic arrangement of *vipR* and insecticidal protein genes within the canonical BtPAI-1 and two representative BtPAI-1 variants. The canonical BtPAI-1 is represented by strain HD-1, whereas the BtPAI-1 variants are represented by strains BGSC 4J5 and HD73, both of which lack *vipR*. Heterologous introduction of *vipR* into these strains promoted vegetative-phase transcription of *cry* genes. Arrow orientation indicates gene transcription direction, with right-pointing arrows representing genes located on the positive strand and left-pointing arrows representing genes located on the negative strand. Different colors denote different gene categories, and gene labels indicate the corresponding insecticidal proteins encoded by each locus.
