# Supplementary material for: Bacillus thuringiensis pathogenicity islands encode regulatory circuits controlling insecticidal Cry toxin expression during vegetative growth
Source: PLoS Pathog. 2026 Jul 29;22(7):e1014476. doi: 10.1371/journal.ppat.1014476 (PMC13440803; doi:10.1371/journal.ppat.1014476)
Supplement: Supplementary file 11 [file ppat.1014476.s011.docx]

**S8 Fig. Comparative sequence analysis of BtPAI-1 and representative BtPAI-1 variants.**

Nucleotide sequence comparisons were performed using Easyfig v2.2.5 between the canonical BtPAI-1 from strain HD-1 and representative BtPAI-1 variants from strains BGSC 4J5 and HD73. Regions of sequence similarity are indicated by shaded areas connecting homologous loci. The analysis shows that highly homologous insecticidal protein genes are organized in distinct gene arrangements in different genomic backgrounds. Arrows indicate the positions and orientations of predicted coding sequences.
