# Supplementary material for: Bacillus thuringiensis pathogenicity islands encode regulatory circuits controlling insecticidal Cry toxin expression during vegetative growth
Source: PLoS Pathog. 2026 Jul 29;22(7):e1014476. doi: 10.1371/journal.ppat.1014476 (PMC13440803; doi:10.1371/journal.ppat.1014476)
Supplement: Supplementary file 12 [file ppat.1014476.s012.docx]

**S9 Fig. Detection of *vipR* transcriptional levels in HD-1 at sporulation phase.**

The qRT-PCR analysis of *vipR* in HD-1 was performed at 24 h, 36 h and 48 h. The *gapdh* and *16S rRNA* genes were used as internal reference genes. Data represent the means ± SD (n = 3 biological replicates). Statistical significance was determined using appropriate tests (see Materials and Methods). Significance thresholds: P > 0.05 (ns), ****P < 0.0001.
