# Supplementary material for: Bacillus thuringiensis pathogenicity islands encode regulatory circuits controlling insecticidal Cry toxin expression during vegetative growth
Source: PLoS Pathog. 2026 Jul 29;22(7):e1014476. doi: 10.1371/journal.ppat.1014476 (PMC13440803; doi:10.1371/journal.ppat.1014476)
Supplement: Supplementary file 13 [file ppat.1014476.s013.docx]

**S1 Raw Gel. Original Gel for S1 Fig.**

Raw agarose gel electrophoresis image captured using the Smart Gel Imaging System (Model: GV15S-0722-8001) from Guangzhou Biolight Biotechnology Co., Ltd. "del" indicates the mutant gene, and "wt" indicates the wild-type gene.

**S2 Raw Gel. Original Gel for S4 Fig.**

(A) cDNA reverse-transcribed from total RNA of HD-1 was used as the template. Lanes 1–3 represent the verification of the cry2Aa transcription unit using three primer pairs: RT-cry2Aa-1-F/R, RT-cry2Aa-2-F/R, and RT-cry2Aa-3-F/R, respectively. Lanes 4–6 represent the verification of the cry2Ab transcription unit using three primer pairs: RT-cry2Ab-1-F/R, RT-cry2Ab-2-F/R, and RT-cry2Ab-3-F/R, respectively. Lanes 7–10 represent the verification of the amidase transcription unit using three primer pairs: RT-*ami*-1-F/R, RT-*ami*-2-F/R, RT-*cry1Aa*-1-F/R, RT-*cry1Aa*-2-F/R and RT-*cry1Aa*-3-F/R, respectively. Lanes 11–12 represent the verification of the cry2Aa transcription unit using three primer pairs: RT-cry1Ia-1-F/R and RT-cry1Ia-2-F/R, respectively. (B) Genomic DNA (gDNA) was included as positive controls. Raw agarose gel electrophoresis image captured using the Smart Gel Imaging System (Model: GV15S-0722-8001) from Guangzhou Biolight Biotechnology Co., Ltd.

**S3 Raw Gel. Original Gel for S3 Fig.**

Raw agarose gel electrophoresis image captured using the Smart Gel Imaging System (Model: GV15S-0722-8001) from Guangzhou Biolight Biotechnology Co., Ltd. "del" indicates the mutant gene, and "wt" indicates the wild-type gene.

**S4 Raw Gel. Original blot for Fig 1C..**

The marker used was Thermo Scientific PageRuler Prestained Protein Ladder (#26616). Lane 1: HD-1, 9 h culture; Lane 2: HD-1Δ*vipR*, 9 h culture; Lane 3: HD-1, 24 h culture; Lane 4: HD-1Δ*vipR*, 24 h culture; Lane 5: HD-1, 36 h culture; Lane 6: HD-1Δ*vipR*, 36 h culture. Each sample was prepared from the fermentation broth.

**S5 Raw Gel. Original blot for Fig 5E.**

The marker used was Thermo Scientific PageRuler Prestained Protein Ladder (#26616). (A) Lane 1: BGSC 4J5, 9 h culture; Lane 2: BGSC 4J5 (*vipR*), 9 h culture; Lane 3: BGSC 4J5, 24 h culture; Lane 4: BGSC 4J5 (*vipR*), 24 h culture; Lane 5: BGSC 4J5, 36 h culture; Lane 6: BGSC 4J5 (*vipR*), 36 h culture. (A) Lane 1: HD73, 9 h culture; Lane 2: HD73 (*vipR*), 9 h culture; Lane 3: HD73, 24 h culture; Lane 4: HD73 (*vipR*), 24 h culture; Lane 5: HD73, 36 h culture; Lane 6: HD73 (*vipR*), 36 h culture. Each sample was prepared from the fermentation broth.

**S6 Raw Gel. Original Gel for Fig 2I-2L.**

EMSA analysis of VipR binding to the promoter fragments of *amidase*, *cry2Ab*, *cry1Ia*, and *cry2Aa*. (A) The *amidase* promoter fragment was used as the probe. Lane 1: no VipR protein (negative control); Lane 2: +150 nM VipR; Lane 3: +250 nM VipR; Lane 4: +350 nM VipR; Lane 5: +350 nM VipR + 100-fold excess unlabeled probe (competition assay); Lane 6: +350 nM VipR + mutant probe (VipR binding site deleted). (B) The *cry2Ab* promoter fragment was used as the probe. Lane 1: no VipR protein (negative control); Lane 2: +150 nM VipR; Lane 3: +250 nM VipR; Lane 4: +350 nM VipR; Lane 5: +350 nM VipR + 100-fold excess unlabeled probe (competition assay); Lane 6: +350 nM VipR + mutant probe (VipR binding site deleted). (C) The *cry1Ia* promoter fragment was used as the probe. Lane 1: no VipR protein (negative control); Lane 2: +50 nM VipR; Lane 3: +150 nM VipR; Lane 4: +250 nM VipR; Lane 5: : +350 nM VipR; Lane 6:+350 nM VipR + 100-fold excess unlabeled probe (competition assay); Lane 7: +350 nM VipR + mutant probe (VipR binding site deleted). (D) The *cry2Aa* promoter fragment was used as the probe. Lane 1: no VipR protein (negative control); Lane 2: +150 nM VipR; Lane 3: +250 nM VipR; Lane 4: +350 nM VipR; Lane 5: +350 nM VipR + 100-fold excess unlabeled probe (competition assay); Lane 6: +350 nM VipR + mutant probe (VipR binding site deleted).
