# Supplementary material for: Bacillus thuringiensis pathogenicity islands encode regulatory circuits controlling insecticidal Cry toxin expression during vegetative growth
Source: PLoS Pathog. 2026 Jul 29;22(7):e1014476. doi: 10.1371/journal.ppat.1014476 (PMC13440803; doi:10.1371/journal.ppat.1014476)
Supplement: Supplementary file 14 [file ppat.1014476.s014.docx]

**Reviewers' Comments:**

Reviewer's Responses to Questions

**Part I - Summary**
Please use this section to discuss strengths/weaknesses of study, novelty/significance, general execution and scholarship.

**Reviewer #1:**

This is a solid and interesting study that, in my view, is suitable for publication after minor revision. The manuscript presents convincing evidence that VipR binds promoter regions and promotes vegetative-phase expression of several toxin genes in the strains examined. Furthermore, the distribution of VipR across Bt genomes reported in the manuscript supports the view that this may represent a broadly relevant regulatory mechanism within *Bacillus thuringiensis*. The work is mechanistically relevant and adds useful insight into phase-specific regulation of insecticidal gene expression in *Bacillus thuringiensis*.

**Response:** We thank reviewer **# 1** for the positive and constructive evaluation of our manuscript. We are pleased that the reviewer found the study to be solid and scientifically interesting, and consider that the data provide convincing evidence for the role of VipR in the regulation of toxin gene expression and its broader relevance in *Bacillus thuringiensis*. We also are grateful for the recognition that our work provides novel mechanistic insights into the phase-specific regulation of insecticidal gene expression in *B. thuringiensis*.

**Reviewer #2:**

Most cry genes depend on the sporulation-specific σᴱ/σᴷ regulatory cascade, leading to substantial Cry protein accumulation primarily during sporulation. In this work, the authors identify VipR, a BtPAI-1-encoded transcriptional regulator, as a key activator of *cry* gene expression during the vegetative phase. It uncovers a previously unrecognized regulatory mechanism that controls *cry* gene expression during the vegetative phase and revises the current model of Cry protein regulation. Moreover, it demonstrates that BtPAI-1 functions as a mobile genetic unit that encodes both insecticidal genes and the regulatory circuits that coordinate insecticidal protein production.

**Response:** We thank the reviewer for the insightful and positive evaluation of our work. We are pleased that the reviewer recognizes VipR as a key activator of *cry* gene expression during the vegetative phase and appreciates the significance of this previously unrecognized regulatory mechanism. We are also grateful for the recognition that BtPAI-1 functions as a mobile genetic element that not only carries insecticidal genes but also encodes the regulatory circuit required to coordinate their expression.

**Reviewer #3:**

In summary, this paper reports evidence of a regulatory program, particularly turning on virulence factors against target insects, that are motile along pathegenicity islands. The evidence supports that these virulence factors are beyond those sigma factors that are essential to sporulation and that coincidentally also drive expression of those virulence factors during sporulation. The evidence supports the genetic mobility and its linkages to particular virulence factors. Importantly, this new regulatory protein drives vegetative expression, increasing the often crystalline accumulation of virulence factors within wild-type Bt. This genetically mobile transcriptional regulator is found in various types of wild-type Bt, including several that have been characterized (i.e. HD-1). The strengths of the paper include it's clear writing, thoroughness of experimental exploration for the claims made, and generalizability of impact. While not entirely novel, as there are at least two papers out on VipR, the novelty of *vipR* shared among independent groups, is indicative of its early significance while I think will only expand over time. I find no conceptual weakness and few technical weaknesses, which I'll describe in detail in Part III.

**Response:** We thank the reviewer for the thoughtful and positive evaluation of our work. We greatly appreciate the recognition of the significance of the regulatory program described in this study, particularly its role in controlling expression of insecticidal virulence factors through a genetically mobile regulatory system associated with BtPAI-1-related elements. We are also grateful for comments on the broader distribution of VipR across *Bacillus thuringiensis* strains, including well-characterized isolates, which supports the general relevance and potential impact of our findings.

As noted, VipR has been previously reported as a regulator of Vip3Aa expression. In this study, we extend its regulatory framework by demonstrating its direct role in the activation of *cry* gene expression during the vegetative phase and linking it to a mobile genetic element, thereby providing new insights into the phase-specific regulation and the coordinated control of insecticidal factors.

**Part II – Major Issues: Key Experiments Required for Acceptance**
Please use this section to detail the key new experiments or modifications of existing experiments that should be absolutely required to validate study conclusions.

Generally, there should be no more than 3 such required experiments or major modifications for a "Major Revision" recommendation. If more than 3 experiments are necessary to validate the study conclusions, then you are encouraged to recommend "Reject".

**Reviewer #1:**

I did not identify any major issues that would preclude publication after minor revision.

**Response:** We thank the reviewer for its positive evaluation of our manuscript.

**Reviewer #2:**

1. Line 176-178 “This pronounced reduction in insecticidal activity correlated with the diminished accumulation of Vip3Aa and Cry proteins in HD-1Δ*vipR* during the vegetative phase. But it is not clear the respective proportions of Vip3A and Cry in virulence. The expression of *vip3A* peaks in the vegetative phase, and Vip3A may exert mainly insecticidal activity in the fermentation broth of HD-1. To determine whether the reduced Cry accumulation in the *vipR* mutant affected insecticidal activity during the vegetative phase, the most suitable control strain should be ∆*vip3A*. This mutant strain knocks out *vip3A* while retaining *vipR*, eliminating interference from Vip3A.

**Response:** Thank you very much for this insightful and constructive suggestion. We fully agree that because Vip3Aa is a major vegetative-phase insecticidal protein, it is important to distinguish its contribution from that of Cry proteins when interpreting the reduced insecticidal activity observed in the *vipR* mutant.

To address this concern, we constructed a *vip3Aa* deletion mutant (HD-1Δ*vip3Aa*) in the HD-1 background using the CRISPR-Cas9 system. We then compared the insecticidal activities of HD-1, HD-1Δ*vipR*, and HD-1Δ*vip3Aa* during both vegetative and sporulation phases. These results have been incorporated into the revised manuscript (Fig. 1D and Fig. 1E) (Revised version, marked: Lines 208-212; unmarked Lines 175-219).

During the vegetative phase (9 h), the fermentation broth of wild-type HD-1 caused 80.1% ± 2.9% larval mortality, whereas HD-1Δ*vipR* exhibited a markedly reduced mortality rate of 12.5% ± 1.4%. In contrast, HD-1Δ*vip3Aa* retained significantly higher insecticidal activity than HD-1Δ*vipR*, causing 26.4% ± 1.4% mortality, although this value remained substantially lower than that of wild-type HD-1. These results indicate that the severe reduction in early insecticidal activity caused by *vipR* deletion cannot be attributed solely to the loss of Vip3Aa expression. Rather, the additional decrease observed in HD-1Δ*vipR* is consistent with the reduced accumulation of Cry proteins during the vegetative phase (Revised version, marked: Lines 214-235; unmarked Lines 175-200).

We further determined the LC_50_ values of spore-crystal mixtures from the three strains. Wild-type HD-1 exhibited an LC_50_ value of 2.23 μg/g (95% confidence interval [CI]: 1.65–3.14 μg/g), whereas HD-1Δ*vipR* displayed a significantly elevated LC_50_ value of 31.74 μg/g (95% CI: 24.02–38.62 μg/g). The HD-1Δ*vip3Aa* strain exhibited an intermediate LC_50_ value of 22.72 μg/g (95% CI: 17.94–28.78 μg/g), consistent with previous reports showing that deletion of *vip3Aa* reduces insecticidal activity. Although HD-1Δ*vip3Aa* appeared somewhat more toxic than HD-1Δ*vipR*, the overlap between their 95% confidence intervals indicates that this difference was not statistically significant. We speculate that the partial recovery of Cry production during sporulation may compensate for the reduced vegetative-phase Cry accumulation observed in the *vipR* mutant (Revised version, marked: Lines 236-250; unmarked Lines 201-215).

Together, these additional experiments directly address the reviewer’s concern and support the conclusion that VipR contributes to insecticidal activity not only through regulation of Vip3Aa expression, but also through promotion of Cry protein accumulation during vegetative growth.

2. In Fig 2, Panel I, J, K, and L, non-specific competitor (poly(dI-dC)) or unlabeled mutation probes (core binding sequence has been mutated) should be added to exclude non-specific binding. In addition, in panel L, why is there a lack of a 200-fold cold probe competition assay for *cry2A*?

**Response:** We thank the reviewer for these constructive comments and for carefully examining our EMSA data presented in Fig. 2. We appreciate reviewer’s important suggestions, which have helped us to further strengthen the rigor and clarity of our binding assays.

In response to the concern regarding potential non-specific binding in Fig. 2I-2L, we repeated the EMSA experiments using the promoter regions of *cry2Ab*, *amidase*, *cry1Ia* and *cry2Aa* (P*_cry2Ab_*, P*_amidase_*, P*_cry1Ia_*, and P*_cry2Aa_*). In the revised assays, we included 100 ng of poly(dI-dC) as a non-specific competitor in the binding reactions. In addition, we generated mutant probes in which the predicted VipR core binding motifs were deleted. As shown in Fig. 2I-2L, VipR binding was effectively abolished when the mutated probes were used, demonstrating that the observed interactions depend on the integrity of the predicted binding sequence and confirming the specificity of VipR-DNA recognition (Revised version, marked: Lines 340-352; unmarked Lines 303-314).

Regarding the absence of a 200-fold cold probe competition assay for *cry2Aa* promoter (P*_cry2Aa_*) in the original Fig. 2L, we sincerely apologize for this omission. We have now repeated the EMSA experiments and included the complete competition assay in the updated figure. The revised data consistently demonstrate that addition of excess unlabeled probe effectively competed for binding, further supporting specific interaction between VipR and the P*_cry2Aa_* promoter region (new Fig. 2L) (Revised version, marked: Lines 340-352; unmarked Lines 303-314).

We thank the reviewer again for this insightful comment, which has enabled us to provide additional experimental evidence supporting the specificity of VipR binding and significantly improving the overall quality of the manuscript.

**Reviewer #3:**

I have no major issues, but I do think that the method of protein quantification is Necessary to add in the methods.

**Response:** We thank the reviewer for this helpful suggestion. We apologize for the omission of this information in the original Methods section. We have revised the manuscript to provide a clear and detailed description of how parasporal crystal-spore preparations were quantified and normalized prior to insect bioassays. Briefly, parasporal crystal-spore mixtures produced after different growth times were collected and resuspended to a defined volume, and samples were standardized based on equivalent spore counts. These samples were used for mortality assays such as figure 1D. For LC_50_ values determination these strains were grown for 60 h and spore-crystal samples were recovered, washed and freeze-dried. The lyophilized preparations were resuspended at 1000 μg/mL and serially diluted to final concentrations of 100, 10, 5, 2.5, 1.25 and 0.625 μg/mL. These suspensions were mixed with artificial diet for insect bioassays and LC_50_ values determinations. The LC_50_ values of bioassays were reported as μg/g of diet. These methodological details have now been included in the revised manuscript (Revised version, marked: Lines 866-904; unmarked Lines 799-831).

**Part III – Minor Issues: Editorial and Data Presentation Modifications**
Please use this section for editorial suggestions as well as relatively minor modifications of existing data that would enhance clarity.

**Reviewer #1:**

Major points within a minor revision

1. Some conclusions are overstated and should be restricted to the strains and targets directly examined.
The manuscript occasionally generalizes the proposed two-phase regulatory model too broadly. In my view, the data support such a model for the strains and promoters that were experimentally tested. However, extension of this framework to all strains carrying VipR-binding motifs or related BtPAI-1 architectures should be phrased more cautiously as a hypothesis or broader model, rather than as a demonstrated general principle.

**Response:** We thank the reviewer for this important and constructive comment. We agree that our current data directly support the proposed two-phase regulatory model only for the strains and promoters that were specifically examined.

Accordingly, we have carefully revised the manuscript to avoid overgeneralization and to more clearly distinguish between experimentally supported conclusions and broader hypotheses. Throughout the text, we have modified the relevant statements regarding the regulatory role of VipR to clearly indicate in which strains the VipR may regulate which insecticidal protein genes containing VipR-binding motifs, rather than implying that this mechanism operates across all Bt strains or all BtPAI-1-related genetic elements.

In addition, we reconsidered our previous classification of BtPAI-1-associated elements into multiple subtypes. Although our comparative genomic analyses identified several structurally distinct incomplete BtPAI-1 architectures, functional validation was performed only in two representative strains (HD73 and BGSC 4J5) lacking *vipR*. To better reflect the scope of our experimental evidence and avoid implying functional conservation across all previously defined subtypes, we have simplified the classification throughout the manuscript. We now distinguish between (i) the canonical BtPAI-1 and (ii) BtPAI-1 variants, which collectively encompass incomplete or structurally rearranged BtPAI-1-related elements. Accordingly, our conclusions regarding VipR function have been restricted to the representative BtPAI-1 variants that were directly examined in this study.

These revisions have been incorporated throughout the manuscript to ensure that our interpretations remain appropriately supported by the available data and that broader implications are presented as hypotheses requiring further experimental validation (Revised version, marked: Lines 32-58, Lines 60-83, Lines 149, Lines 156-159, Lines 202-203, Lines 338, Lines 351, Lines 402, Lines 421, Lines 452-471, Lines 481, Lines 475, Lines 488, Lines 490, Lines 495, Lines 497, Lines 523, Lines 531-532, Lines 535, Lines 575, Lines 591, Lines 597-598, Lines 634-635, Lines 638, Lines 639-640, Lines 645-646, Lines 656 and Lines 739-741; unmarked Lines 32-47, Lines 24-47, Lines 131, Lines 134-135, Lines 138-140, Lines 274, Lines 301, Lines 313-314, Lines 361-362, Lines 381, Lines 412-430, Lines 439, Lines 445, Lines 447, Lines 454, Lines 479, Lines 487-490, Lines 529, Lines 544, Lines 550-551, Lines 587-588, Lines 591-593, Lines 598-599, Lines 609, Lines 692-694).

2. The temporal annotation throughout the manuscript needs to be harmonized carefully.
There are several inconsistencies in reported sampling time points across the Results, figure panels, figure legends, and Materials and Methods. Because the manuscript’s central conclusions depend on phase-specific expression and protein accumulation, this is not a minor editorial detail. The authors should systematically reconcile all time-point information and ensure that each figure and corresponding description refer to the same experimental design and sampling scheme.

Response: We sincerely thank the reviewer for this careful and important observation. We fully agree that accurate and consistent temporal annotation is essential for interpretation of phase-specific gene expression, protein accumulation and overall conclusions of the study.

In response, we conducted a comprehensive review of all time point information across the Results, figure panels, figure legends, and Materials and Methods. We now systematically corrected and harmonized the reported sampling times and corrected all inconsistencies to ensure that the experimental design and sampling scheme are described consistently across all sections of the manuscript. All discrepancies have been corrected. In addition, we revised the Results section to clearly indicate the sampling time points associated with the qRT-PCR analyses, western blot experiments and microscopic observation wherever relevant. Corresponding corrections and clarifications have been incorporated into the manuscript to ensure complete consistency between the presented data and their descriptions (Fig 1A-C, Fig 2A-2D, Fig 3A-3B, Fig 4A-4D, Fig 4E) (Revised version, marked: Lines 175-185, Lines 214-218, Lines 225-228, Lines 230-231, Line 259, Lines 266-267, Lines 302, Lines 305, Lines 308 Lines 310, Lines 394, Lines 397, Lines 399-400, Lines 430, Lines 501, Lines 503-504 and Lines 511; unmarked Lines 155-166, Lines 182-185, Lines 191-196, Lines 223-224, Lines 266-269, Lines 271-274, Lines 354-362, Lines 462, Lines 495, Lines 619).

3. The manuscript would benefit from a clearer mechanistic presentation of VipR promoter interactions.
The authors convincingly show promoter binding and positive effects on transcript accumulation, but the mechanistic interpretation remains underdeveloped. In particular, it would be highly informative to include a simple schematic for one or a few representative promoters showing the approximate position of the VipR-binding motif relative to the -35/-10 elements, the transcription start site (if known), and the start codon. This would help the reader assess plausible regulatory mechanisms and seems a reasonable addition based on the promoter and motif analyses already presented.

**Response:** We thank the reviewer for this helpful and constructive suggestion. We agree that a clearer schematic representation of the promoter architecture would facilitate interpretation of the potential mechanism by which VipR regulates transcription.

In the revised manuscript, we have included a new supplementary figure (Fig S5) that schematically illustrates representative VipR-regulated promoters. This figure shows the relative positions of the predicted VipR-binding motif, the putative -35 and -10 promoter elements, and the translational start codon.

Since transcription start sites have not been experimentally determined (e.g., by 5′ RACE or primer extension), they are not included in the schematic figure.

**Reviewer #2:**

1. Line 306-308. “These results indicate that increased *vipR* expression promotes the transcription of multiple toxin genes across diverse Bt genetic backgrounds.” This conclusion may be premature here. Only two strains (HD-1 and CT-43) have been tested, and both harbor BtPAI-1. Thus, evidence for “diverse Bt genetic backgrounds” is insufficient. The authors may draw this conclusion in Line 450, after determining the transcription level of the *cry9Aa* gene in strain BGSC 4AE1 with introduced *vipR*.

**Response:** We fully agree with the reviewer that the conclusion should be restricted to the strains directly tested in this study. Since only two Bt strains (HD-1 and CT-43), both of which harbor BtPAI-1, were analyzed in the current work, we agree that it is premature to generalize the observed effect to diverse genetic backgrounds.

Accordingly, we have revised the text of the manuscript to avoid overgeneralization and to ensure that conclusions accurately reflect the scope of the experimental evidence. In addition, as suggested by the reviewer, we now include the analysis of *cry9Aa* transcription in strain BGSC 4AE1 following introduction of *vipR*. Based on these results, we now state that VipR may promote the transcription of additional toxin genes containing putative VipR-binding motifs across other Bt genetic backgrounds outside the specific genetic contexts represented by HD-1 and CT-43, rather than presenting this as a universally established mechanism. The corresponding changes have been incorporated into the revised manuscript (Revised version, marked: Lines 401-402 and Lines 597-598; unmarked Lines 360-362, Lines 489-490).

2. Line 80 " Lepidoptera, Coleoptera" should be in regular font.

**Response:** We have corrected the formatting in the revised manuscript, and “Lepidoptera” and “Coleoptera” are now presented in regular font. (Revised version, marked: Lines 102; unmarked Lines 84)

Reviewer #3:

I have some minor issues, none of which are absolutely required to fix but would help clarify things for the reader, if addressed. One throughout is font size in some of the Figures. This can be troubling, I know. Perhaps the less relevant genes, the IS elements and functional genes other than ICPs and VIPs, don't have to be labeled within the Supplemental Figure 5, for example. They aren't pertinent to the findings or conclusions.

**Response:** We thank the reviewer for these helpful suggestions. We have carefully reviewed all figures and standardized font sizes throughout the manuscript to improve consistency and readability.

In addition, several new supplementary figures were added during the revision process. Consequently, the original Fig. S5 has been renumbered as new Fig. S7. We have revised this new Fig. S7 by removing the names of IS elements and other genes that are not directly relevant to ICP and Vip proteins. These modifications simplify the figure and allow us to focus the figure on the key findings of the study.

Going through the manuscript, line 168 "equivalent cell numbers" is confusing. Cell numbers or counts of parasporal crystals are not counted. The line refers to Figure 1C, which is a Western blot which I find hard to believe that equivalent cell numbers were scored or loaded into these lanes. This brings me to my next small issue, which I honestly have with any manuscript that quantifies protein-spore mixes for biological assays: concentration and normalization of cells/proteins for input into assays and gels. LC_50_s are recorded as ug/g, but how protein concentration was determined is never mentioned. This part is important. If, for example, ∆*vipR* cells generate lower Cry concentrations, is this compensated for an increase in spores per culture volume? If BCA analysis was used, spores might confound protein measurements due to reducing sugars. Or perhaps other extracellular proteins are upregulated. When normalized for a protein level for an assay or a gel, are there more spores then? This is complicated as it's possible that the LC_50_ would appear to decrease if some other protein were made in compensation for the loss of vegetative synthesis of VIPs and ICPs. But given that ICPs are the major product in the broth that is lost in ∆*vipR* strains, there is an increase in its LC_50_, reflective of a decrease in the expression of known virulence factors. One could imagine a scenario in which total Crys were instead quantitated, and the LC_50_ (if cultures were normalized to ug/g Cry content) might not change, but in that case, one would have an increase in spores to get the same ug/g of Cry proteins. At the very least, I would need to know how protein concentrations were measured, how inputs were normalized, so we can reflect on what that means given the complexities of a spore crystal lysate.

**Response:** We thank the reviewer for this detailed and insightful comment regarding normalization and quantification in spore–crystal preparations. We fully agree that accurate interpretation of LC_50_ values in such systems critically depends on the normalization strategy used.

First, in the experiment shown in Fig 1C, samples were collected at the vegetative (9 h), the early sporulation (24 h) and the latesporulation (36 h) phases. Cell density of each sample was determined using the dilution plate counting method. Then these samples were normalized to 1 × 10^7^ CFU/mL. The Western Blots were loaded with similar number of cells (2 × 10^5^ CFU), with equal volumes of 20 μl loaded per lane. We clearly explained this procedure and have therefore revised the text (Revised version marked: Line 912-922; unmarked Lines 839-849)

Second, we appreciate the reviewer´s discussion of the challenges associated with quantifying and normalizing spore-crystal preparations. In this study, we did not normalize samples based on total protein content, as protein-based quantification in spore–crystal mixtures can be confounded by the presence of spores and other cellular components as the reviewer pointed out. Instead, we standardized the preparations based on equivalent spore numbers that were lyophilized to obtain spore/crystal powders, then these materials were resuspended at 1000 μg/mL and serially diluted to final concentrations of 100, 10, 5, 2.5, 1.25 and 0.625 μg/mL. This approach ensures that the LC_50_ values reflect insecticidal activity of comparable biological material ratherthan protein-normalized toxin concentrations. (Revised version marked: Line 867-904; unmarked Lines 800-826)

We have now clarified this normalization strategy in the Materials and Methods section to improve transparency and avoid potential confusion.

**Methods:** Spore–crystal mixtures were harvested and resuspended to a defined volume. The cell density of the washed sample was determined using the dilution plate counting method, and all samples were adjusted to a uniform density (1 × 10^7^ CFU/mL) with sterile ddH2O. The normalized samples were then lyophilized using a freeze dryer to obtain spore-crystal powders (Life Technologies). The lyophilized preparations were resuspended at 1000 μg/mL and serially diluted to final concentrations of 100, 10, 5, 2.5, 1.25 and 0.625 μg/mL. These suspensions were mixed with artificial diet for insect bioassays and LC_50_ values determinations.

3 mL of each diluted sample was thoroughly mixed with 15 g of artificial diet [59]. The mixed diet was transferred to 24-well culture plates, with one newly hatched neonate S. frugiperda larva per well. Each sample was performed in triplicate, and mortality was recorded after seven days. The LC50 values were calculated using probit analysis (Revised version, marked: Lines 867-904; unmarked Lines 800-826).

Finally, Figure 1A: at a glance, these images do not seem support the claim that there is a difference in parasporal association with crystals. Instead, it looks like the ∆*vipR* strain expresses Cry proteins earlier since the 30 h mark is the most prevalently stained while HD-1 Crystals aren'0t obvious until 42 h. I would zoom in further to show that the ∆*vipR* strain has many spores without crystals, a qualitative zoom-in of WHAT exactly is quantified in Figure 1B. These are minor issues.

**Response:** We thank the reviewer for this careful and constructive comment. We agree that the original Fig 1A did not optimally illustrate the specific phenotype quantified in Fig 1B.

To address this concern, we have revised Fig 1A and improved the presentation of these images. In the updated figure, we have added higher-magnification (zoom-in) panels highlighting representative fields, which better illustrate spores lacking crystal inclusions, particularly in the HD-1Δ*vipR* strain. Fig S2 also confirmed these observations. We have also replaced the original images with higher quality images to improve comparability among strains and time points (Fig 1A and Fig S2).

We also carefully considered the reviewer’s observation that images could be interpreted as showing earlier crystal formation in the HD-1Δ*vipR* strain. Upon re-evaluation, we concluded that visual assessment of crystal-associated spores during the early and intermediate stages of sporulation (24–36 h) is inherently challenging because spores and crystal inclusions are not yet fully separated, and they can be difficult to distinguish reliably. Consequently, these images were not ideally suited for supporting quantitative comparisons at those time points (24-36 h). Therefore, to ensure a more robust and objective analysis, we revised the quantification strategy and restricted the crystal-to-spore ratio measurements to the late sporulation stage (42 h), when spores and crystal inclusions are clearly distinguishable. This approach minimizes potential classification errors and provides a more reliable basis for comparing crystal formation between strains.

We believe these modifications have significantly improved both the clarity of the microscopy data presented in Fig 1A and the rigor of the quantitative analysis shown in Fig 1B (Revised version, marked: Lines 175-185; unmarked Lines 155-166).
