# Supplementary material for: Bacillus thuringiensis pathogenicity islands encode regulatory circuits controlling insecticidal Cry toxin expression during vegetative growth
Source: PLoS Pathog. 2026 Jul 29;22(7):e1014476. doi: 10.1371/journal.ppat.1014476 (PMC13440803; doi:10.1371/journal.ppat.1014476)
Supplement: Peer review history

# Peer review: Decision Letter 0

**DOI:** 10.1371/journal.ppat.1014476.r001

**Authors:** Alice Prince, Raffi V. Aroian

16 Apr 2026

-->PPATHOGENS-D-26-00779

Bacillus thuringiensis pathogenicity islands encode regulatory circuits controlling insecticidal Cry toxin expression during vegetative growth.

PLOS Pathogens

Dear Dr. Peng,

Thank you for submitting your manuscript to PLOS Pathogens. After careful consideration, we feel that it has merit but does not fully meet PLOS Pathogens's publication criteria as it currently stands. Therefore, we invite you to submit a revised version of the manuscript that addresses the points raised during the review process.

Please submit your revised manuscript by Jun 15 2026 11:59PM. If you will need more time than this to complete your revisions, please reply to this message or contact the journal office at plospathogens@plos.org. When you're ready to submit your revision, log on to https://www.editorialmanager.com/ppathogens/ and select the 'Submissions Needing Revision' folder to locate your manuscript file.

Please include the following items when submitting your revised manuscript:

* A letter that responds to each point raised by the editor and reviewer(s). You should upload this letter as a separate file labeled 'Response to Reviewers'. This file does not need to include responses to any formatting updates and technical items listed in the 'Journal Requirements' section below.

* A marked-up copy of your manuscript that highlights changes made to the original version. You should upload this as a separate file labeled 'Revised Manuscript with Track Changes'.

* An unmarked version of your revised paper without tracked changes. You should upload this as a separate file labeled 'Manuscript'.

If you would like to make changes to your financial disclosure, competing interests statement, or data availability statement, please make these updates within the submission form at the time of resubmission. Guidelines for resubmitting your figure files are available below the reviewer comments at the end of this letter.

We look forward to receiving your revised manuscript.

Kind regards,

Raffi V. Aroian

Academic Editor

PLOS Pathogens

Alice Prince

Section Editor

PLOS Pathogens

Sumita Bhaduri-McIntosh

Editor-in-Chief

PLOS Pathogens

orcid.org/0000-0003-2946-9497

Michael Malim

-->Editor-in-Chief

PLOS Pathogens

orcid.org/0000-0002-7699-2064

**Additional Editor Comments :**

Two reviewers were enthusiastic about the manuscript with minor/no revisions. One reviewer has asked for some important control experiments that seem relevant. Please address all comments.

**Journal Requirements:**

1) We ask that a manuscript source file is provided at Revision. Please upload your manuscript file as a .doc, .docx, .rtf or .tex. If you are providing a .tex file, please upload it under the item type u2018LaTeX Source Fileu2019 and leave your .pdf version as the item type u2018Manuscriptu2019.

2) Please upload all main figures as separate Figure files in .tif or .eps format. For more information about how to convert and format your figure files please see our guidelines:

https://journals.plos.org/plospathogens/s/figures

3) We have noticed that you have uploaded Supporting Information files, but you have not included a list of legends. Please add a full list of legends for your Supporting Information files after the references list.

4) Please amend your detailed Financial Disclosure statement. This is published with the article. It must therefore be completed in full sentences and contain the exact wording you wish to be published.

1) State what role the funders took in the study. If the funders had no role in your study, please state: "The funders had no role in study design, data collection and analysis, decision to publish, or preparation of the manuscript."

5) Some material included in your submission may be copyrighted. According to PLOS’s copyright policy, authors who use figures or other material (e.g., graphics, clipart, maps) from another author or copyright holder must demonstrate or obtain permission to publish this material under the Creative Commons Attribution 4.0 International (CC BY 4.0) License used by PLOS journals. Please closely review the details of PLOS’s copyright requirements here: PLOS Licenses and Copyright. If you need to request permissions from a copyright holder, you may use PLOS's Copyright Content Permission form.

Please respond directly to this email and provide any known details concerning your material's license terms and permissions required for reuse, even if you have not yet obtained copyright permissions or are unsure of your material's copyright compatibility. Once you have responded and addressed all other outstanding technical requirements, you may resubmit your manuscript within Editorial Manager.

Potential Copyright Issues:

Figure 7: Please confirm whether you drew the images / clip-art within the figure panels by hand. If you did not draw the images, please provide (a) a link to the source of the images or icons and their license / terms of use; or (b) written permission from the copyright holder to publish the images or icons under our CC BY 4.0 license. Alternatively, you may replace the images with open source alternatives. See these open source resources you may use to replace images / clip-art:

- https://commons.wikimedia.org

- https://openclipart.org/.

Note: If the reviewer comments include a recommendation to cite specific previously published works, please review and evaluate these publications to determine whether they are relevant and should be cited. There is no requirement to cite these works unless the editor has indicated otherwise.

**Reviewers' Comments:**

Reviewer's Responses to Questions

**Part I - Summary**

Please use this section to discuss strengths/weaknesses of study, novelty/significance, general execution and scholarship.

Reviewer #1: This is a solid and interesting study that, in my view, is suitable for publication after minor revision. The manuscript presents convincing evidence that VipR binds promoter regions and promotes vegetative-phase expression of several toxin genes in the strains examined. Furthermore, the distribution of VipR across Bt genomes reported in the manuscript supports the view that this may represent a broadly relevant regulatory mechanism within Bacillus thuringiensis. The work is mechanistically relevant and adds useful insight into phase-specific regulation of insecticidal gene expression in Bacillus thuringiensis.

Reviewer #2: Most cry genes depend on the sporulation-specific σE/σK regulatory cascade, leading to substantial Cry protein accumulation primarily during sporulation. In this work, the authors identify VipR, a BtPAI-1-encoded transcriptional regulator, as a key activator of cry gene expression during the vegetative phase. It uncovers a previously unrecognized regulatory mechanism that controls cry gene expression during the vegetative phase and revises the current model of Cry protein regulation. Moreover, it demonstrates that BtPAI-1 functions as a mobile genetic unit that encodes both insecticidal genes and the regulatory circuits that coordinate insecticidal protein production.

Reviewer #3: In summary, this paper reports evidence of a regulatory program, particularly turning on virulence factors against target insects, that are motile along pathegenicity islands. The evidence supports that these virulence factors are beyond those sigma factors that are essential to sporulation and that coincidentally also drive expression of those virulence factors during sporulation. The evidence supports the genetic mobility and its linkages to particular virulence factors. Importantly, this new regulatory protein drives vegetative expression, increasing the often crystalline accumulation of virulence factors within wild-type Bt. This genetically mobile transcriptional regulator is found in various types of wild-type Bt, including several that have been characterized (i.e. HD1). The strengths of the paper include it's clear writing, thoroughness of experimental exploration for the claims made, and generalizability of impact. While not entirely novel, as there are at least two papers out on VipR, the novelty of vipR shared among independent groups, is indicative of its early significance while I think will only expand over time. I find no conceptual weakness and few technical weaknesses, which I'll describe in detail in Part III.

**********

**Part II – Major Issues: Key Experiments Required for Acceptance**

Please use this section to detail the key new experiments or modifications of existing experiments that should be absolutely required to validate study conclusions.

Generally, there should be no more than 3 such required experiments or major modifications for a "Major Revision" recommendation. If more than 3 experiments are necessary to validate the study conclusions, then you are encouraged to recommend "Reject".

Reviewer #1: I did not identify any major issues that would preclude publication after minor revision.

Reviewer #2: 1. Line 176-178 “This pronounced reduction in insecticidal activity correlated with the diminished accumulation of Vip3Aa and Cry proteins in HD-1ΔvipR during the vegetative phase. ” But it is not clear the respective proportions of Vip3A and Cry in virulence. The expression of vip3A peaks in the vegetative phase, and Vip3A may exert mainly insecticidal activity in the fermentation broth of HD-1. To determine whether the reduced Cry accumulation in the vipR mutant affected insecticidal activity during the vegetative phase, the most suitable control strain should be ∆vip3A. This mutant strain knocks out vip3A while retaining vipR, eliminating interference from Vip3A.

2. In Fig 2, Panel I, J,K, and L, non-specific competitor (poly(dI-dC)) or unlabeled mutation probes (core binding sequence has been mutated) should be added to exclude non-specific binding. In addition, in panel L, why is there a lack of a 200-fold cold probe competition assay for cry2A?

Reviewer #3: I have no major issues, but I do think that the method of protein quantification is Necessary to add in the methods.

**********

**Part III – Minor Issues: Editorial and Data Presentation Modifications**

Please use this section for editorial suggestions as well as relatively minor modifications of existing data that would enhance clarity.

Reviewer #1: Major points within a minor revision

1. Some conclusions are overstated and should be restricted to the strains and targets directly examined.

The manuscript occasionally generalizes the proposed two-phase regulatory model too broadly. In my view, the data support such a model for the strains and promoters that were experimentally tested. However, extension of this framework to all strains carrying VipR-binding motifs or related BtPAI-1 architectures should be phrased more cautiously as a hypothesis or broader model, rather than as a demonstrated general principle.

2. The temporal annotation throughout the manuscript needs to be harmonized carefully.

There are several inconsistencies in reported sampling time points across the Results, figure panels, figure legends, and Materials and Methods. Because the manuscript’s central conclusions depend on phase-specific expression and protein accumulation, this is not a minor editorial detail. The authors should systematically reconcile all time-point information and ensure that each figure and corresponding description refer to the same experimental design and sampling scheme.

3. The manuscript would benefit from a clearer mechanistic presentation of VipR promoter interactions.

The authors convincingly show promoter binding and positive effects on transcript accumulation, but the mechanistic interpretation remains underdeveloped. In particular, it would be highly informative to include a simple schematic for one or a few representative promoters showing the approximate position of the VipR-binding motif relative to the -35/-10 elements, the transcription start site (if known), and the start codon. This would help the reader assess plausible regulatory mechanisms and seems a reasonable addition based on the promoter and motif analyses already presented.

Reviewer #2: 1. Line 306-308. “These results indicate that increased vipR expression promotes the transcription of multiple toxin genes across diverse Bt genetic backgrounds.” This conclusion may be premature here. Only two strains (HD-1 and CT-43) have been tested, and both harbor BtPAI-1. Thus, evidence for “diverse Bt genetic backgrounds” is insufficient. The authors may draw this conclusion in Line 450, after determining the transcription level of the cry9Aa gene in strain BGSC 4AE1 with introduced vipR.

2. Line 80 " Lepidoptera, Coleoptera" should be in regular font.

Reviewer #3: I have some minor issues, none of which are absolutely required to fix but would help clarify things for the reader, if addressed. One throughout is font size in some of the Figures. This can be troubling, I know. Perhaps the less relevant genes, the IS elements and functional genes other than ICPs and VIPs, don't have to be labeled within the Supplemental Figure 5, for example. They aren't pertinent to the findings or conclusions. Going through the manuscript, line 168 "equivalent cell numbers" is confusing. Cell numbers or counts of parasporal crystals are not counted. The line refers to Figure 1C, which is a Western blot which I find hard to believe that equivalent cell numbers were scored or loaded into these lanes. This brings me to my next small issue, which I honestly have with any manuscript that quantifies protein-spore mixes for biological assays: concentration and normalization of cells/proteins for input into assays and gels. LC50s are recorded as ug/g, but how protein concentration was determined is never mentioned. This part is important. If, for example, ∆vipR cells generate lower Cry concentrations, is this compensated for an increase in spores per culture volume? If BCA analysis was used, spores might confound protein measurements due to reducing sugars. Or perhaps other extracellular proteins are upregulated. When normalized for a protein level for an assay or a gel, are there more spores then? This is complicated as it's possible that the LC50 would appear to decrease if some other protein were made in compensation for the loss of vegetative synthesis of VIPs and ICPs. But given that ICPs are the major product in the broth that is lost in ∆vipR strains, there is an increase in its LC50, reflective of a decrease in the expression of known virulence factors. One could imagine a scenario in which total Crys were instead quantitated, and the LC50 (if cultures were normalized to ug/g Cry content) might not change, but in that case, one would have an increase in spores to get the same ug/g of Cry proteins. At the very least, I would need to know how protein concentrations were measured, how inputs were normalized, so we can reflect on what that means given the complexities of a spore crystal lysate. Finally, Figure 1A: at a glance, these images do not seem support the claim that there is a difference in parasporal association with crystals. Instead, it looks like the ∆vipR strain expresses Cry proteins earlier since the 30h mark is the most prevalently stained while HD-1 Crystals aren't obvious until 42h. I would zoom in further to show the that the ∆vipR strain has many spores without crystals, a qualitative zoom-in of WHAT exactly is quantified in Figure 1B. These are minor issues.

**********

PLOS authors have the option to publish the peer review history of their article (what does this mean?). If published, this will include your full peer review and any attached files.

If you choose “no”, your identity will remain anonymous but your review may still be made public.

**Do you want your identity to be public for this peer review?**  For information about this choice, including consent withdrawal, please see our Privacy Policy.

Reviewer #1: **Yes:**  Dr. Heiko Liesegang

Reviewer #2: No

Reviewer #3: No

[NOTE: If reviewer comments were submitted as an attachment file, they will be attached to this email and accessible via the submission site. Please log into your account, locate the manuscript record, and check for the action link "View Attachments". If this link does not appear, there are no attachment files.]

**Figure resubmission:**

-->While revising your submission, we strongly recommend that you use PLOS’s NAAS tool (https://ngplosjournals.pagemajik.ai/artanalysis) to test your figure files. NAAS can convert your figure files to the TIFF file type and meet basic requirements (such as print size, resolution), or provide you with a report on issues that do not meet our requirements and that NAAS cannot fix.

After uploading your figures to PLOS’s NAAS tool - https://ngplosjournals.pagemajik.ai/artanalysis, NAAS will process the files provided and display the results in the "Uploaded Files" section of the page as the processing is complete. If the uploaded figures meet our requirements (or NAAS is able to fix the files to meet our requirements), the figure will be marked as "fixed" above. If NAAS is unable to fix the files, a red "failed" label will appear above. When NAAS has confirmed that the figure files meet our requirements, please download the file via the download option, and include these NAAS processed figure files when submitting your revised manuscript.

**Reproducibility:**

To enhance the reproducibility of your results, we recommend that authors of applicable studies deposit laboratory protocols in protocols.io, where a protocol can be assigned its own identifier (DOI) such that it can be cited independently in the future. Additionally, PLOS ONE offers an option to publish peer-reviewed clinical study protocols. Read more information on sharing protocols at https://plos.org/protocols?utm_medium=editorial-email&utm_source=authorletters&utm_campaign=protocols

# Author comment: Author response to Decision Letter 1

**DOI:** 10.1371/journal.ppat.1014476.r002

13 Jun 2026

Attachment Submitted filename: Response to Reviewers.docx

# Peer review: Decision Letter 1

**DOI:** 10.1371/journal.ppat.1014476.r003

**Authors:** Alice Prince, Raffi V. Aroian

16 Jul 2026

Dear Dr. Peng,

We are pleased to inform you that your manuscript ' Bacillus thuringiensis pathogenicity islands encode regulatory circuits controlling insecticidal Cry toxin expression during vegetative growth.' has been provisionally accepted for publication in PLOS Pathogens.  You have addressed all significant comments and all reviewers, including myself, believe this manuscript provides important research ready to publish.

Before your manuscript can be formally accepted you will need to complete some formatting changes, which you will receive in a follow up email. A member of our team will be in touch with a set of requests.

Please note that your manuscript will not be scheduled for publication until you have made the required changes, so a swift response is appreciated.

IMPORTANT: The editorial review process is now complete. PLOS will only permit corrections to spelling, formatting or significant scientific errors from this point onwards. Requests for major changes, or any which affect the scientific understanding of your work, will cause delays to the publication date of your manuscript.

Should you, your institution's press office or the journal office choose to press release your paper, you will automatically be opted out of early publication. We ask that you notify us now if you or your institution is planning to press release the article. All press must be co-ordinated with PLOS.

Thank you again for supporting Open Access publishing; we are looking forward to publishing your work in PLOS Pathogens.

Best regards,

Raffi V. Aroian

Academic Editor

PLOS Pathogens

Alice Prince

Section Editor

PLOS Pathogens

Sumita Bhaduri-McIntosh

Editor-in-Chief

PLOS Pathogens

orcid.org/0000-0003-2946-9497

Michael Malim

Editor-in-Chief

PLOS Pathogens

orcid.org/0000-0002-7699-2064

***********************************************************

Reviewer Comments (if any, and for reference):

Reviewer's Responses to Questions

**Part I - Summary**

Please use this section to discuss strengths/weaknesses of study, novelty/significance, general execution and scholarship.

Reviewer #1: The study represents a solid study that strengthens the field.

Thanks the authors for carefully addressing all comments raised during the first review round. The revised manuscript has been substantially improved, and the conclusions are now appropriately aligned with the experimental evidence presented. I have no further concerns and support publication of the manuscript in its current form.

Reviewer #2: In this work, the authors identify VipR, a BtPAI-1-encoded transcriptional regulator, as a key activator of cry gene expression during the vegetative phase. It uncovers a previously unrecognized regulatory mechanism that controls cry gene expression during the vegetative phase and revises the current model of Cry protein regulation. Moreover, it demonstrates that BtPAI-1 functions as a mobile genetic unit that encodes both insecticidal genes and the regulatory circuits that coordinate insecticidal protein production.

**********

**Part II – Major Issues: Key Experiments Required for Acceptance**

Please use this section to detail the key new experiments or modifications of existing experiments that should be absolutely required to validate study conclusions.

Generally, there should be no more than 3 such required experiments or major modifications for a "Major Revision" recommendation. If more than 3 experiments are necessary to validate the study conclusions, then you are encouraged to recommend "Reject".

Reviewer #1: I had no major issues in my review of the original manuscript.

Reviewer #2: The authors have comprehensively revised the manuscript point by point in accordance with all the revision comments and requirements put forward in the previous review. All raised questions have been responded to clearly and reasonably, and the revisions have effectively addressed the deficiencies existing in the original manuscript.

**********

**Part III – Minor Issues: Editorial and Data Presentation Modifications**

Please use this section for editorial suggestions as well as relatively minor modifications of existing data that would enhance clarity.

Reviewer #1: Reviewer #1 considers all previously raised concerns to be adequately addressed. The authors have appropriately limited the generalization of VipR function to the genetic contexts directly investigated in this study, harmonized temporal annotations throughout the manuscript, and revised several interpretations to more closely align with the experimental evidence presented.

Reviewer #2: There are no additional revision comments or concerns for this manuscript.

**********

PLOS authors have the option to publish the peer review history of their article (what does this mean?). If published, this will include your full peer review and any attached files.

If you choose “no”, your identity will remain anonymous but your review may still be made public.

**Do you want your identity to be public for this peer review?**  For information about this choice, including consent withdrawal, please see our Privacy Policy.

Reviewer #1: No

Reviewer #2: No

# Editor report: Acceptance letter

**DOI:** 10.1371/journal.ppat.1014476.r004

**Authors:** Alice Prince, Raffi V. Aroian

Dear Dr. Peng,

We are delighted to inform you that your manuscript, " Bacillus thuringiensis pathogenicity islands encode regulatory circuits controlling insecticidal Cry toxin expression during vegetative growth.," has been formally accepted for publication in PLOS Pathogens.

We have now passed your article onto the PLOS Production Department who will complete the rest of the pre-publication process. All authors will receive a confirmation email upon publication.

The corresponding author will soon be receiving a typeset proof for review, to ensure errors have not been introduced during production. Please review the PDF proof of your manuscript carefully, as this is the last chance to correct any scientific or type-setting errors. Please note that major changes, or those which affect the scientific understanding of the work, will likely cause delays to the publication date of your manuscript. Note: Proofs for Front Matter articles (Pearls, Reviews, Opinions, etc...) are generated on a different schedule and may not be made available as quickly.

Soon after your final files are uploaded, the early version of your manuscript, if you opted to have an early version of your article, will be published online. The date of the early version will be your article's publication date. The final article will be published to the same URL, and all versions of the paper will be accessible to readers.

For Research Articles, you will receive an invoice from PLOS for your publication fee after your manuscript has reached the completed accept phase. If you receive an email requesting payment before acceptance or for any other service, this may be a phishing scheme. Learn how to identify phishing emails and protect your accounts at https://explore.plos.org/phishing.

Thank you again for supporting open-access publishing; we are looking forward to publishing your work in PLOS Pathogens.

Best regards,

Sumita Bhaduri-McIntosh

Editor-in-Chief

PLOS Pathogens

orcid.org/0000-0003-2946-9497

Michael Malim

Editor-in-Chief

PLOS Pathogens

orcid.org/0000-0002-7699-2064
